# Retrieval Interference in Syntactic Processing: The Case of Reflexive Binding in English

**DOI:** 10.3389/fpsyg.2016.00329

**Published:** 2016-05-26

**Authors:** Umesh Patil, Shravan Vasishth, Richard L. Lewis

**Affiliations:** ^1^Department of Linguistics, University of PotsdamPotsdam, Germany; ^2^Computational Linguistics, Institute of Cognitive Science, University of OsnabrückOsnabrück, Germany; ^3^Department of Psychology, University of MichiganAnn Arbor, MI, USA

**Keywords:** sentence processing, anaphor resolution, memory retrieval, interference, computational modeling, eye tracking

## Abstract

It has been proposed that in online sentence comprehension the dependency between a reflexive pronoun such as *himself/herself* and its antecedent is resolved using exclusively syntactic constraints. Under this *strictly syntactic search* account, Principle A of the binding theory—which requires that the antecedent c-command the reflexive within the same clause that the reflexive occurs in—constrains the parser's search for an antecedent. The parser thus ignores candidate antecedents that might match agreement features of the reflexive (e.g., gender) but are ineligible as potential antecedents because they are in structurally illicit positions. An alternative possibility accords no special status to structural constraints: in addition to using Principle A, the parser also uses non-structural cues such as gender to access the antecedent. According to cue-based retrieval theories of memory (e.g., Lewis and Vasishth, 2005), the use of non-structural cues should result in increased retrieval times and occasional errors when candidates partially match the cues, even if the candidates are in structurally illicit positions. In this paper, we first show how the retrieval processes that underlie the reflexive binding are naturally realized in the Lewis and Vasishth (2005) model. We present the predictions of the model under the assumption that both structural and non-structural cues are used during retrieval, and provide a critical analysis of previous empirical studies that failed to find evidence for the use of non-structural cues, suggesting that these failures may be Type II errors. We use this analysis and the results of further modeling to motivate a new empirical design that we use in an eye tracking study. The results of this study confirm the key predictions of the model concerning the use of non-structural cues, and are inconsistent with the strictly syntactic search account. These results present a challenge for theories advocating the infallibility of the human parser in the case of reflexive resolution, and provide support for the inclusion of agreement features such as gender in the set of retrieval cues.

## 1. Introduction

Sentence comprehension involves, among other things, recovering hierarchical structure from an input string of words (e.g., Frazier, 1979). Such recovery requires the online application of grammatical constraints that delimit the possible relationships between various elements of the sentence. For example, to understand a sentence like (1), the pronoun *himself* has to be resolved to a referent of an earlier noun *surgeon*; the reflexive cannot be associated with *Jonathan* due to Principle A of the binding theory (Chomsky, 1981)^1^.

(1) The surgeon who treated Jonathan had pricked himself.

Establishing a relationship between two non-adjacent elements in a sentence requires maintaining some memory of the immediate past. The question we are concerned with here is: what role do grammatical and non-grammatical constraints play in the access to the immediate past? The binding of English reflexive pronouns is a particularly informative case, because the configurational and agreement constraints are relatively clear, and the structure admits manipulations of distance and distracting candidate antecedents (Sturt, 2003).

One proposal for how structural constraints are implicated in dependency resolution is motivated by the experiments reported in Nicol and Swinney (1989), Sturt (2003), and Xiang et al. (2009). Using different experimental methodologies, these studies found that a grammatically incorrect antecedent [e.g., *Jonathan* in (1)] does not interfere in the process of binding a reflexive pronoun by a grammatically correct antecedent [e.g., *surgeon* in (1)], at least in the early stages of processing a reflexive.

Nicol and Swinney (1989) presented evidence from a series of experiments that employed the cross modal lexical priming paradigm. Participants listened to sentences similar to those shown in (2) and responded to visually presented probe words that was presented immediately following the reflexive *himself*. The probe word was either semantically related or unrelated to one of the three previously occurring nouns in the sentence (*boxer, skier*, or *doctor*). Participants judged whether the probe word was a word or non-word. A significant priming effect was observed when probe words were related to grammatically accessible as antecedents [e.g., *doctor* in (2)], but not when they were related to grammatically inaccessible antecedents [e.g., *boxer* and *skier* in (2)]. Nicol and Swinney (1989) concluded that no priming was observed for words related to grammatically inaccessible antecedents because they had not been considered during co-reference resolution.

(2) The boxer told the skier that the doctor for the team would blame himself for the recent injury.

Sturt (2003) reported eye tracking studies using materials such as (3). He found that first fixation duration and first pass reading time on the region containing the reflexive were longer when the gender of the reflexive did not match the stereotypical gender of the grammatically accessible antecedent than when it matched (e.g., *herself* and *surgeon* vs. *himself* and *surgeon*). Early reading times were *not* affected by gender match between the reflexive and the grammatically inaccessible antecedent (*Jonathan* or *Jennifer*). Second pass reading time at the reflexives showed an interaction for gender match between the reflexive and the two antecedents suggesting that in later interpretation stages (but, crucially, not in earlier processing stages)^2^ the inaccessible antecedent is part of the candidates being considered as antecedents. There was also an effect of the inaccessible antecedent in second pass reading time in the pre-final region, but this effect was observed only when the accessible antecedent matched the gender of the reflexive. However, these late effects of the inaccessible antecedent were not observed in the second experiment [with design as in (4)], but he pointed out that the absence of any effect of the inaccessible antecedent in Experiment 2 could have been due to the fact that the inaccessible antecedent did not c-command the reflexive and it was also not as prominent as in Experiment 1.

To gain further insight into this late-stage interpretation of the sentences, Sturt (2003) also ran a follow-up study, where a sentence-by-sentence self-paced reading task was followed by a question that directly probed for the antecedent of the reflexive. This study showed a significant interference effect, with more ungrammatical interpretations when the grammatically inaccessible antecedent matched the gender of the reflexive; the effect was bigger when the accessible antecedent did not match the gender of the reflexive. Sturt (2003) concluded that grammatical constraints are applied very early in processing, but interference from the grammatically inaccessible antecedent occurs during later processes that are related to recovery strategies, rather than during processes related to the initial interpretation of the reflexive.

(3) {Jonathan/Jennifer} was pretty worried at the City Hospital. {He/She} remembered that the surgeon had pricked {himself/herself} with a used syringe needle. There should be an investigation soon.
(4) {Jonathan/Jennifer} was pretty worried at the City Hospital. The surgeon who treated {Jonathan/Jennifer} had pricked {himself/herself} with a used syringe needle. There should be an investigation soon.

Dillon et al. (2013) reported two eye tracking studies with English reflexives using material with syntactic structure similar to Experiment 2 in Sturt (2003). They also did not find any effect of the inaccessible antecedent, but reported effects of the accessible antecedent. Xiang et al. (2009) reported similar results in an ERP study, where they found that a P600 is elicited by a reflexive that mismatches the stereotypical gender of the grammatically accessible antecedent, and is not attenuated by the presence of a matching antecedent in a grammatically inaccessible position.

Based on results from these studies, Phillips et al. (2011) suggests that:

“…*argument reflexives are immune to interference from structurally inaccessible antecedents because antecedents are retrieved using only structural cues. In effect, we are suggesting that the person, gender, and number features of reflexives like himself, herself, and themselves play no role in the search for antecedents …”*

An alternative possibility accords no special status to the structural constraints: in addition to using Principle A, the parser also uses non-structural cues such as gender to access the antecedent. For example, in (1), it is possible that the parser considers a relation between *Jonathan* and *himself*, due to a gender-feature match, and perhaps also due to the relative proximity of *Jonathan* compared to *surgeon*. Under at least one cue-based retrieval theory (Lewis and Vasishth, 2005; Lewis et al., 2006), this should result in interference from grammatically inaccessible antecedent while resolving the reflexive-antecedent dependency^3^. Some evidence for this account comes from studies reported in Badecker and Straub (2002), Choy and Thompson (2010), and Cunnings and Felser (2013) among others.

Badecker and Straub (2002) reported an interference effect from gender-matched distractors in a self-paced reading experiment using sentences as in (5). They found that reading times two words beyond a reflexive were slowed by the presence of a gender matching NP in a grammatically inaccessible position.

(5) {Jane/John} thought that Bill owed himself another opportunity to solve the problem.

More evidence for early retrieval interference in reflexive binding comes from the study reported in Cunnings and Felser (2013). In two eye tracking studies they tested how application of Principle A varies between low and high working memory span readers. In the first study they found a late effect of inaccessible antecedent, emerging only at regions following the reflexive region. However, in the second study where the inaccessible antecedent was closer in the surface string to the reflexive, the effect of inaccessible antecedent was observed in an early eye movement measure, namely first fixation duration, at the reflexive itself, although this effect was limited to low span readers. Consequently, Cunnings and Felser (2013) conclude that “lower span participants were more likely to consider both potential antecedents of the reflexive early on during processing, before converging on the structurally accessible antecedent later on.”

Further evidence for the effect of interference from grammatically inaccessible antecedent comes from an eye tracking study in a visual world paradigm reported by Choy and Thompson (Thompson and Choy, 2009; Choy and Thompson, 2010). Although this study was targeted at aphasics' processing deficits with binding constructions, for present purposes we consider data only from unimpaired participants. Choy and Thompson (2010) recorded eye movements while the participants listened to a story as in (6), with the critical sentence containing a pronoun or a reflexive (e.g., *him* or *himself*). The visual stimuli consisted of pictures of two persons, one of which was grammatically accessible and the other inaccessible (e.g., *soldier* and *farmer*); a human-referring distractor; and an inanimate-referring noun mentioned in the story (e.g., *glasses*). The data for the reflexive condition from unimpaired participants showed an increase in the proportion of fixations to the inaccessible antecedent in the reflexive and post-reflexive regions compared to the pre-reflexive region. Although the proportion of fixations to the accessible antecedent was higher than the fixations to the inaccessible antecedent in most of the regions, the increase in fixations to the inaccessible antecedent from the onset of the reflexive indicates that participants considered the inaccessible antecedent as the potential antecedent of the reflexive, albeit less often than the accessible antecedent.

(6) Some soldiers and farmers were in a house. The soldier told the farmer with glasses to shave {himself/him} in the bathroom. And he did.

In summary, the effect of interference from a grammatically inaccessible antecedent is sometimes observed in early processing and sometimes in late processing, and in some studies the effect is completely absent.

In the remainder of this paper, we first apply an existing computational model of cue-based parsing (Lewis and Vasishth, 2005) to an empirical paradigm well established for testing the processing of reflexives in English. The model generates qualitative predictions and demonstrates that these predictions are robust against substantial variation in the quantitative parameters. We then use the theoretical perspective provided by the model to formulate conjectures for why some of the existing empirical work may have failed to detect evidence for the use of non-structural cues. Based on this analysis we advance a new experimental design which is intended to be more sensitive, and demonstrate that for many of the predictions the modified design yields larger effects in modeling simulations. We next present an eye tracking study based on the modified design, yielding several results that confirm the early use of non-structural cues in a manner consistent with the model. The paper concludes with discussion of the implication of these new results for some current theoretical approaches to dependency resolution.

## 2. Modeling reflexive binding in the cue-based retrieval framework

The cue-based retrieval architecture provides a natural characterization of the retrieval steps triggered in the process of reflexive resolution. We begin by presenting a model of Experiment 1 and its follow-up in Sturt (2003), which will provide insight into the predicted effects and their robustness against parametric variation, and provide motivation for the modified design used in the eye tracking study reported here. The emphasis of the model described here is not on parsing the entire sentence, but on detailed modeling of the retrieval process carried out at the reflexive.

Experiment 1 in Sturt (2003) included an eye tracking experiment in which participants were required to read short texts consisting of three sentences. An example is given in (7), showing the four experimental conditions. A named referent (*Jonathan* or *Jennifer*) is introduced in the first sentence, and this referent is subsequently referred to using a pronoun (*he* or *she*) in the second sentence. The second sentence also introduces a second referent *the surgeon*, and includes a reflexive anaphor (*himself* or *herself*). The first named referent is not a grammatically accessible antecedent for the reflexive in terms of binding theory, while the second referent (*the surgeon*) is a grammatically accessible antecedent. Accessible and inaccessible antecedents either matched or did not match the gender of the reflexive. Note that even when the accessible antecedent doesn't match the gender of the reflexive, the sentences are still grammatical because a *surgeon* is only stereotypically masculine and hence a licit antecedent of *herself*.

In match-interference and match conditions [(a) and (b) in (7)] the accessible antecedent matches the gender requirement of the reflexive, and in mismatch-interference and mismatch conditions [(c) and (d) in (7)] it does not. Furthermore, in match-interference and mismatch-interference conditions the inaccessible antecedent matches the gender of the reflexive. Henceforth, we will refer to match-interference and match conditions simply as *match* conditions, and mismatch-interference and mismatch conditions as *mismatch* conditions, reflecting the fact that the accessible antecedent matches the gender of the reflexive for one pair and does not for the other. We will refer to match-interference and mismatch-interference conditions as the *interference* conditions because the gender of the inaccessible antecedent matches that of the reflexive—potentially causing interference.

(7) **Sentence 1:** {Jonathan/Jennifer} was pretty worried at the City Hospital.
  **Sentence 2:**
  a. *Accessible-match/inaccessible-match* (*Match-interference*)
    He remembered that the surgeon had pricked **himself** with a used syringe needle.
  b. *Accessible-match/inaccessible-mismatch* (*Match*)
    She remembered that the surgeon had pricked **himself** with a used syringe needle.
  c. *Accessible-mismatch/inaccessible-match* (*Mismatch-interference*)
    She remembered that the surgeon had pricked **herself** with a used syringe needle.
  d. *Accessible-mismatch/inaccessible-mismatch* (*Mismatch*)
    He remembered that the surgeon had pricked **herself** with a used syringe needle.
  **Sentence 3:** There should be an investigation soon.

**Figure 1:**
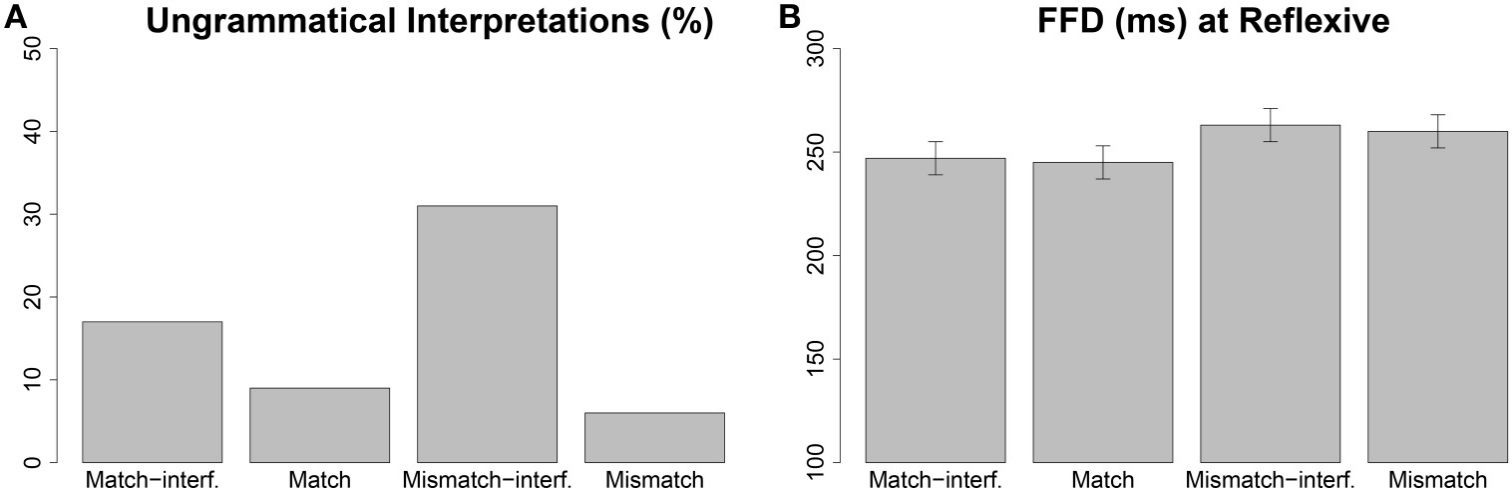
**The effects in Sturt (2003) Experiment 1: (A) proportions of ungrammatical interpretations of reflexives in the follow-up study; (B) first fixation durations in the eye tracking study**. The error bars in the plot for first fixation duration show 95% confidence intervals.

This eye tracking study showed an early effect of the accessible antecedent (Figure 1). First fixation duration and first pass reading time were faster in the match conditions compared to the mismatch conditions. But no effect of the inaccessible antecedent was found in the early measures. The effect of inaccessible antecedent was found only in later measures—second pass reading time was shorter in match-interference condition compared to the match condition.

As mentioned above, Sturt (2003) also conducted a follow-up study to find out the participants' final interpretation of the reflexive. This was a sentence-by-sentence self-paced reading with the same sentences as in (7) but, instead of sentence 3, there was a question that explicitly probed for the antecedent of the reflexive [e.g., a question like *Who had been pricked with a used needle?* with possible answers, for example, for condition (a) as *Jonathan* or *surgeon*]. The follow-up study showed a main effect of accessible antecedent, inaccessible antecedent and also an interaction between these two factors (see (Figure 1). When the accessible antecedent did not match the gender of the reflexive, participants made a higher proportion of errors in selecting the correct antecedent. In addition, when the inaccessible antecedent matched the gender of the reflexive participants made more errors than when it did not. Moreover, the increase in error due to gender match with the inaccessible antecedent was larger when the accessible antecedent did not match the gender, resulting in the interaction between the two factors.

Thus, there are four major findings in Sturt's Experiment 1 and his follow-up study. First, gender mismatch with the default gender of the accessible antecedent resulted in lower question-response accuracies. Second, gender match with the inaccessible antecedent resulted in lower question-response accuracies. Third, early reading time measures (first fixation duration and first pass reading time) increased when the gender specification of the accessible antecedent mismatched that of the reflexive. Fourth, second pass reading time (re-reading time) was *shorter* when the gender of the inaccessible antecedent matched the gender of the reflexive (this occurred in the case where the accessible antecedent matched the reflexive in gender, i.e., in match conditions).

Interestingly, the first three of the four effects can be explained by simply assuming that the search for an antecedent includes a gender feature. Therefore, we begin our modeling by assuming that both grammatical knowledge about antecedent and gender matching is used when resolving antecedents in English. For simplicity, we model the grammatical constraint by assuming that the antecedent should be a noun and should be the subject of the clause containing the reflexive, albeit under some different implementation of this grammatical constraint, the predictions may turn out differently^4^. This choice of retrieval cues is motivated by the conjecture that including agreement features in general may be an adaptive feature of the parser, although attempting to establish this is not the purpose of the work reported here. As a result the set of retrieval cues for the reflexives *himself* and *herself* are {gender = masculine/feminine, category = noun, role = subject, clause = current-clause}, differing only in the value of the *gender* feature. See Table 1 for the list of cues matched by the two antecedents across the four conditions in Sturt's experiments.

**Table 1:** **The match of retrieval cues with the accessible and inaccessible antecedents for the four conditions in Sturt's experiments (cat = category)**.

**Conditions**	**Accessible**	**Inaccessible**
a (match-interference)	Gender, cat, role, clause	Gender, cat, role
b (match)	Gender, cat, role, clause	Cat, role
c (mismatch-interference)	Cat, role, clause	Gender, cat, role
d (mismatch)	Cat, role, clause	Cat, role

The accessible antecedent matches all four cues in conditions (a) and (b) (the “match” conditions), but only three cues in conditions (c) and (d) (the “mismatch” conditions), since the stereotypical gender of *surgeon* is masculine, which does not match the gender retrieval cue at *herself* (gender = feminine). The inaccessible antecedent matches three cues (gender, category, role) out of a total of four cues in conditions (a) and (c), and in conditions (b) and (d) it matches two cues (category, role). As a result, interference for retrieving the antecedent will be higher in conditions (a) and (c) (the “interference” conditions) as compared to conditions (b) and (d). Note that the alternative possibility, as suggested by Phillips and colleagues, is that gender plays no role in retrieval; in that case, the cues for the match (a vs. b) and mismatch conditions (c vs. d) would be identical, leading to no interference.

The cue-based retrieval model predicts that similarity-based interference (SBI) arises at the moment of retrieval. SBI in reflexive binding is manifested in terms of delay in retrieval of the correct antecedent or an error in retrieving the correct antecedent. The delay in retrieval of the correct antecedent is a result of the fan assumption (see Equation 3 in Appendix A of Supplementary Material) that reduces the strength of association between a cue and a target as a function of the number of items associated with that cue. Reduced strength of association means reduced activation boost, which produces higher latencies. On the other hand, the error in retrieval of the correct antecedent is a combined result of activation fan and partial match. Reduction in activation boost of the accessible antecedent due to activation spreading, and partial matching between retrieval cues (the second summation component in Equation 1 in Appendix A of Supplementary Material) and any inaccessible antecedents can lead (probabilistically as a function of activation noise) to higher activation of the inaccessible antecedents. As a result, the probability of retrieving the inaccessible antecedent increases. The greater the partial match with inaccessible antecedents, the higher the percentage of errors in retrieving the accessible antecedent.

We model retrieval in sentence 2 from (7); this is the crucial sentence for generating predictions about the reflexive binding process. The predictions of the model are generated by running 1000 simulations for each condition. All model parameters are set to the values that have been used in the previous models from Lewis and Vasishth (2005), Vasishth and Lewis (2006), and Vasishth et al. (2008). A list of all the parameter values that we use is given in Table A1 in Appendix A of Supplementary Material.

The predicted retrieval error percentages accurately capture the pattern found in the Sturt (2003) follow-up study: There is a main effect of accessible antecedent, inaccessible antecedent, and an interaction between these two factors, exactly as in Sturt's follow-up study's response accuracies. First, when the accessible antecedent does not match the gender of the reflexive the model makes a higher number of errors in retrieving the correct antecedent (the mismatch effect in response accuracy). Second, when the inaccessible antecedent matches the gender of the reflexive the model makes more errors than when it does not (the interference effect in response accuracy). Third, the increase in error due to gender match with the inaccessible antecedent is greater in the mismatch conditions.

**Figure 2:**
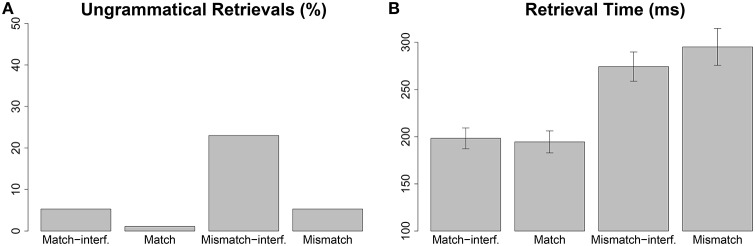
**The twofold predictions of the cue-based retrieval model for Experiment 1 in Sturt (2003), based on the parameter values listed in Table A1 in Supplementary Material. (A)** Proportions of retrievals of the inaccessible antecedent; **(B)** retrieval times at the reflexive. The error bars in the plot for retrieval time show 95% confidence intervals

The retrieval times predicted by the model (shown in Figure 2) show a main effect of matching in the accessible antecedent: When the accessible antecedent does not match the gender of the reflexive, the retrieval times are higher than when it does. The model also predicts a match × interference interaction—retrieval times are predicted to be higher in the *match-interference* condition (198 ms) than in the *match* condition (194 ms); however, retrieval times are predicted to be lower in the *mismatch-interference* condition (274 ms) compared to the *mismatch* condition (295 ms).

In order to compare the predictions to the data, we use the following terminology: the *mismatch effect* is the difference between the *match* conditions and the *mismatch* conditions; the *interference effect* is the effect between the two interference conditions and the other two conditions; the *match-interference effect* is the effect of interference in the two *match* conditions; and the *mismatch-interference effect* is the effect of interference in the two *mismatch* conditions.

The predicted ungrammatical retrievals accurately model the ungrammatical interpretations observed in the Sturt (2003) follow-up study. However, the predicted retrieval times accurately capture only the mismatch effect observed in the first fixation duration (FFD) in the eye tracking study. The interaction predicted between the *mismatch* effect and *interference* effect is not observed in the data. Thus, the model accurately captures the question-response accuracy data, but only partly characterizes the first fixation duration data.

The divergent patterns between the model's retrieval times and the first fixation durations in Sturt's study come from the differences in the patterns seen in the predicted match-interference effect and the mismatch-interference effect. The predicted match-interference effect is a consequence of spreading of activation of the gender cue which is matched by both the accessible and inaccessible antecedent. As described earlier, activation spreading reduces the strength of association between the cue and the target, causing longer retrieval latencies in the match-interference condition than the match condition. On the other hand, the mismatch-interference effect is a consequence of partial match between the cues and inaccessible antecedent: the inaccessible antecedent matches the gender cue which is not matched by the accessible antecedent (see Table 1), leading to higher probability of retrieving the inaccessible antecedent. This can be seen in the predicted retrieval error percentages in Figure 2. Moreover, in the *mismatch-interference* condition the inaccessible antecedent receives more activation from retrieval cues than in the *mismatch* condition as it matches more retrieval cues in the *mismatch-interference* condition. A substantially higher number of incorrect retrievals occur due to higher activation from the retrieval cues, and the retrieval times in the *mismatch-interference* condition are faster than the retrieval times in the *mismatch* condition, contrary to the findings in Sturt's study (see Figure 1 vs. Figure 2). We return to this issue in the Section 3.6.

To summarize, the model predicts the following effects for retrieval errors (RE) and retrieval times (RT) at the reflexive:

E1. Mismatch effect (RE): the retrieval errors for the two mismatch conditions are higher than those for the two match conditions.
E2. Interference effect (RE): the retrieval errors for the match-interference and mismatch-interference conditions are higher than those for the other two conditions.
E3. Mismatch effect (RT): the retrieval times for the two mismatch conditions are longer than those for the two match conditions.
E4. Match-interference effect (RT): the retrieval times for the match-interference condition are longer than the match condition.
E5. Mismatch-interference effect (RT): the retrieval times for the mismatch-interference condition are *shorter* than the mismatch condition.

In Sturt's experiment, only the effects E1, E2 and E3 were observed. The interference effects E4 and E5 were missing in the early measures (first fixation duration and first pass reading time) of the eye tracking studies.

Here we assume that the RE translates to incorrect interpretation of the reflexive and RT translates to reading time in the experiment. We also make a simplified assumption about the lexical representation of nouns with stereotypical gender—as far as the gender feature is concerned, the representation of a stereotypically masculine or feminine noun (e.g., “soldier” or “nurse”) is the same as that of an unambiguously masculine or feminine noun (e.g., “John” or “Jane”). It has been shown that the gender violation effects are stronger for definitionally masculine or feminine nouns than for stereotypically masculine or feminine nouns (Osterhout et al., 1997). This means that our simplified assumption may lead to inflated predictions of various effects than what might be observed in an experiment. Finding out the precise difference in the representation of these two types of nouns is an important research question, but we think it lies outside the scope of this paper. We also assume that the first antecedent that is retrieved, is considered to be the correct antecedent of the reflexive in the final representation irrespective of its gender match with the reflexive; i.e., there is no reanalysis of the reflexive-antecedent dependency.

### 2.1. Parametric variability in the model

**Figure 3:**
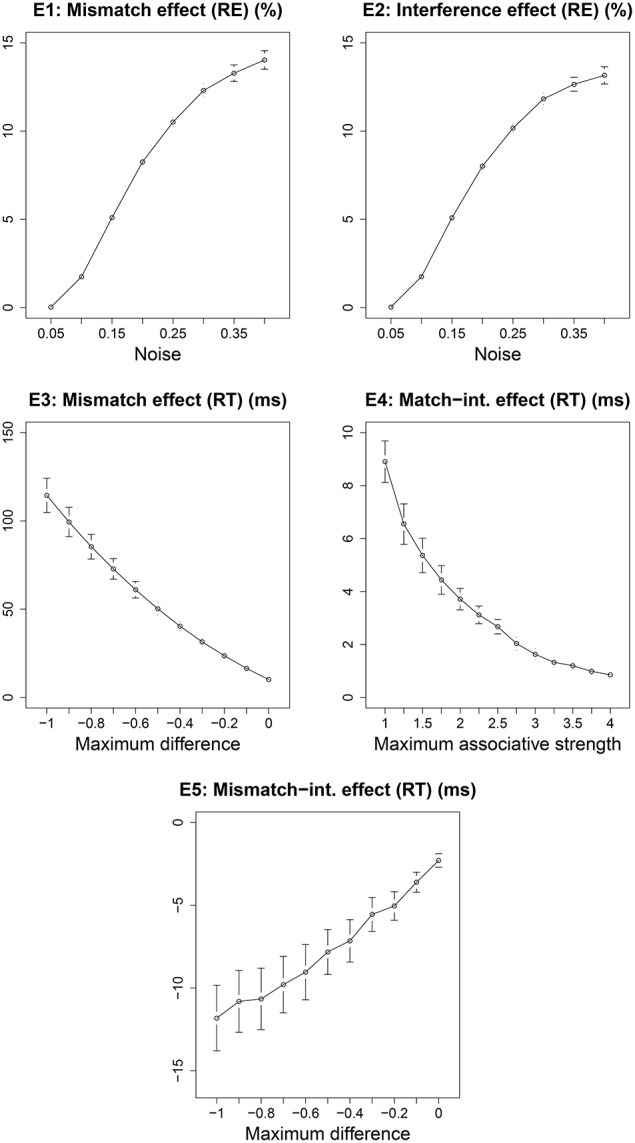
**The predictions of effects E1–E5 for Experiment 1 from Sturt (2003) across a range of parameter values**. Each effect is plotted against the parameter that affects it the most.

We did not estimate any parameter values for the current model. All existing parameters were set to the values that have been used in previous published versions of the cue-based retrieval model. It is possible, however, that the predictions of the model are valid only for the specific parameter values that we used here; this could be the reason behind the lack of effects E4 and E5 in the data—these effects might emerge only for a particular combination of parameter values. Conversely, the correct predictions of effects E1, E2, and E3 might depend on the specific values used by the model. To gain a better understanding of the range of possible predictions of the model, we ran the model for a range of values of three crucial ACT-R parameters: *noise, maximum associative strength* and *maximum difference*. The noise parameter controls the amount of instantaneous activation noise added to each chunk at retrieval; maximum associative strength is the constant “S” in Equation 3 in Supplementary Material; and the maximum difference parameter controls the penalty due to a mismatch between a retrieval cue and a feature value of a chunk. For each of these parameters, the range of values over which the predictions are generated is given in Table 2. The predictions are generated by running 1000 simulations for each combination of values of the three parameters. The total number of combinations of the three parameter values are 1287 (see Table 2). The predictions of effects E1–E5 across these sets of parameter values are plotted in Figure 3. Each effect is plotted against the parameter along which it varies the most. Effects E1 and E2 are influenced the most by noise, E3 and E5 are influenced the most by the maximum difference parameter, and E4 is influenced the most by the maximum associative strength parameter. Each point in the plots represents a mean over all values of the other two parameters. In short, Figure 3 illustrates how the size of each effect varies across different parameter values.

**Table 2:** **The range of parameter values used for testing the parametric variability of the cue-based retrieval models**.

**Parameter**	**Range of values**
Noise	0.05–0.4, in steps of 0.05
Maximum associative strength	1–4, in steps of 0.25
Maximum difference	−1 to 0, in steps of 0.1

The effect E1 varies from 0 to 23.9%, the effect E2 varies from 0 to 17.15%, the effect E3 varies from −1.55 to 228.4 ms, the effect E4 varies from −6.53 to 17.34 ms and the effect E5 varies from from −36.01 to 3.21 ms. The effects E1 and E2 are zero when the instantaneous activation noise is zero, which essentially means that the model does not make any mistake in retrieving the accessible antecedent when there is no noise added to the activations of chunks. But, in general, a non-zero value for noise parameter is necessary for modeling memory errors and response time distribution. Overall, although all effects show variation across different parameter combinations, they all remain mainly non-zero and have the same numerical sign as the predicted effects with the predefined parameter values. These results show that the model's predictions for E1–E3 are not crucially dependent on the fixed parameter values we used.

### 2.2. An alternative explanation for the absence of interference effects (E4 and E5) in Sturt (2003)

Although the lack of an interference effect in Sturt (2003) Experiment 1 could imply that non-structural cues like gender are not used in retrieval, Sturt noticed that the absence of an effect could be due to the non-local linear position of the interferer (inaccessible antecedent) with respect to the reflexive [see (7) above]. The accessible antecedent was introduced later in the string than the inaccessible antecedent, and was therefore closer to the reflexive. In his Experiment 2, Sturt (2003) modified this design by using stimuli as in (8), where the linear positions of the binding accessible and inaccessible antecedents are reversed with relation to Experiment 1, while their accessibility with respect to the binding theory is kept constant. However, this experiment also did not show any interference effect.

(8) {Jonathan/Jennifer} was pretty worried at the City Hospital. The surgeon who treated {Jonathan/Jennifer} had pricked {himself/herself} with a used syringe needle. There should be an investigation soon.

In addition to surface-string locality, we consider now another possibility for the apparent lack of interference: the degree of overlap between potential distractors and retrieval cues. We hypothesized above that reflexive binding uses grammatical category (noun, verb etc.), grammatical role (subject, object etc.) and gender as the retrieval cues to retrieve the correct antecedent. In the cue-based retrieval model, the overlap of these cues with grammatically inaccessible antecedents leads to an interference effect in both retrieval latency and retrieval errors. This formulation in the model leads to the following alternative explanation for the lack of interference effect in Sturt's Experiment 2: the interfering antecedent was the object of the relative clause [see (7) above], and hence did not match the grammatical role cue for retrieval at the reflexive. In fact, Van Dyke and McElree (2011) have recently proposed that although distractors with matching semantic cues exert interference, cues like grammatical role are weighted heavily in the retrieval process. They found that the interference effect due to the semantic match was present only when the distractors matched the grammatical cues as well. These results can also explain the lack of interference effect in Sturt's Experiment 2.

In terms of activations of memory elements, the probability of retrieving an incorrect element is higher if it has a higher activation value at the time of retrieval. The activation value of a memory element is directly dependent on its creation time, retrieval history, and its match with the retrieval cues—the more recently an element is created or retrieved, and the higher feature overlap it has with the retrieval cues, the better chances it has of being retrieved. Consequently, in Sturt's Experiment 1 the interferer has less chance of getting retrieved due to its less recent creation time with respect to the accessible antecedent, and in Experiment 2 the interferer has less chance of getting retrieved because its overlap with the retrieval cues is lower in comparison to the overlap of the accessible antecedent with the retrieval cues. In other words, in Sturt's experiments the inaccessible antecedents may not be strong enough interferers to detect their effect on the retrieval process. If this reasoning is correct, then the effect, or rather the lack of an interference effect, might be a false negative (a type II error). Concluding that an absence of an interference effect is evidence that no interference occurs has important consequences to the theory of retrieval processes in sentence comprehension. No interference in processing argument reflexives implies that the retrieval mechanism for reflexive binding is different from other retrieval mechanisms in sentence processing, e.g., subject-verb agreement, and agreement attraction. On the other hand, finding an interference effect simplifies the theory of retrieval processes considerably, since no exemption is granted to antecedent-reflexive resolution processes.

### 2.3. A modified design

In order to increase the strength of the interference effect, we can use an object relative clause [see (9)] where the inaccessible antecedent has the subject role in the clause. It is also closer to the reflexive in terms of linear distance. Under the cue-based retrieval account, the inaccessible antecedent would be more likely to interfere in the retrieval process than in the two experimental designs in Sturt (2003)—but under the structurally-constrained approach, this manipulation should not matter to the reflexive binding process. In fact, Xiang et al. (2009) used this design in their ERP study, but they did not have the crucial *match* condition. Cunnings and Felser (2013) also used this design to test the interaction of reflexive processing and memory capacity. They do find effect of inaccessible antecedent, but they did not evaluate their findings in terms any specific memory retrieval mechanism. See Section 4 for more details. Note that our design also uses the manipulation of stereotypical gender of the accessible antecedent, as in Sturt (2003), but all sentences are grammatical despite the gender mismatch between the reflexive and the stereotypical gender of the accessible antecedent.

(9)
  a. *Accessible-match/inaccessible-match (match-interference)*
    The tough soldier that Fred treated in the military hospital introduced himself to all the nurses.
  b. *Accessible-match/inaccessible-mismatch (match)*
    The tough soldier that Katie treated in the military hospital introduced himself to all the nurses.
  c. *Accessible-mismatch/inaccessible-match (mismatch-interference)*
    The tough soldier that Katie treated in the military hospital introduced herself to all the nurses.
  d. *Accessible-mismatch/inaccessible-mismatch (mismatch)*
    The tough soldier that Fred treated in the military hospital introduced herself to all the nurses.

We implemented a cue-based retrieval model for the modified design described in (9) as well as for Experiment 2 in Sturt (2003). The goal of this modeling is to compare the predictions of the cue-based retrieval theory for the five effects (E1–E5) across three designs—Experiment 1 (including the follow-up study; we count the eye tracking study and the follow-up study as one experiment, following Sturt), Experiment 2 from Sturt (2003), and the modified design. The modeling assumptions are the same as in the model described above.

**Figure 4:**
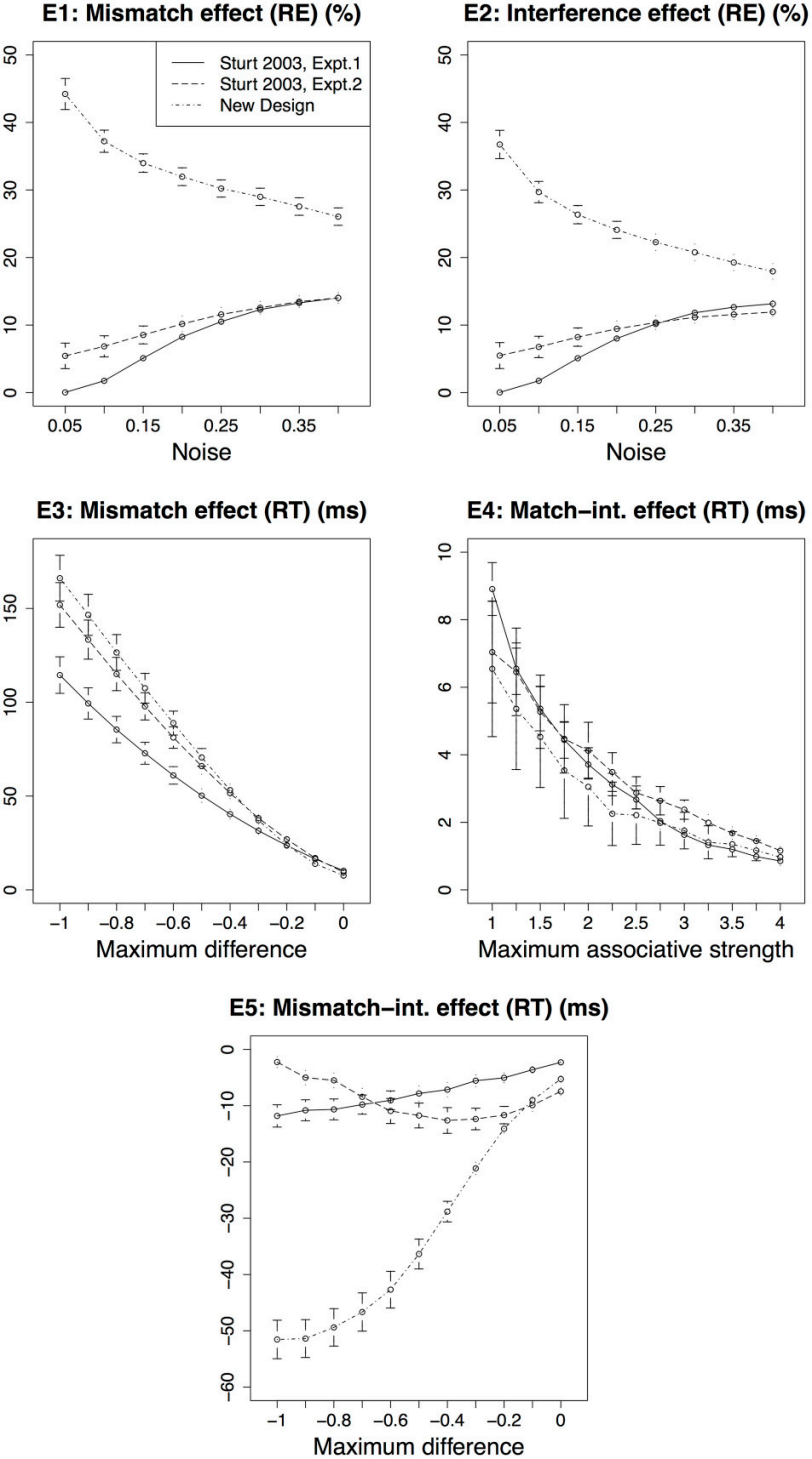
**The predictions of effects E1–E5 for three experimental designs across a range of parameter values**.

**Figure 5:**
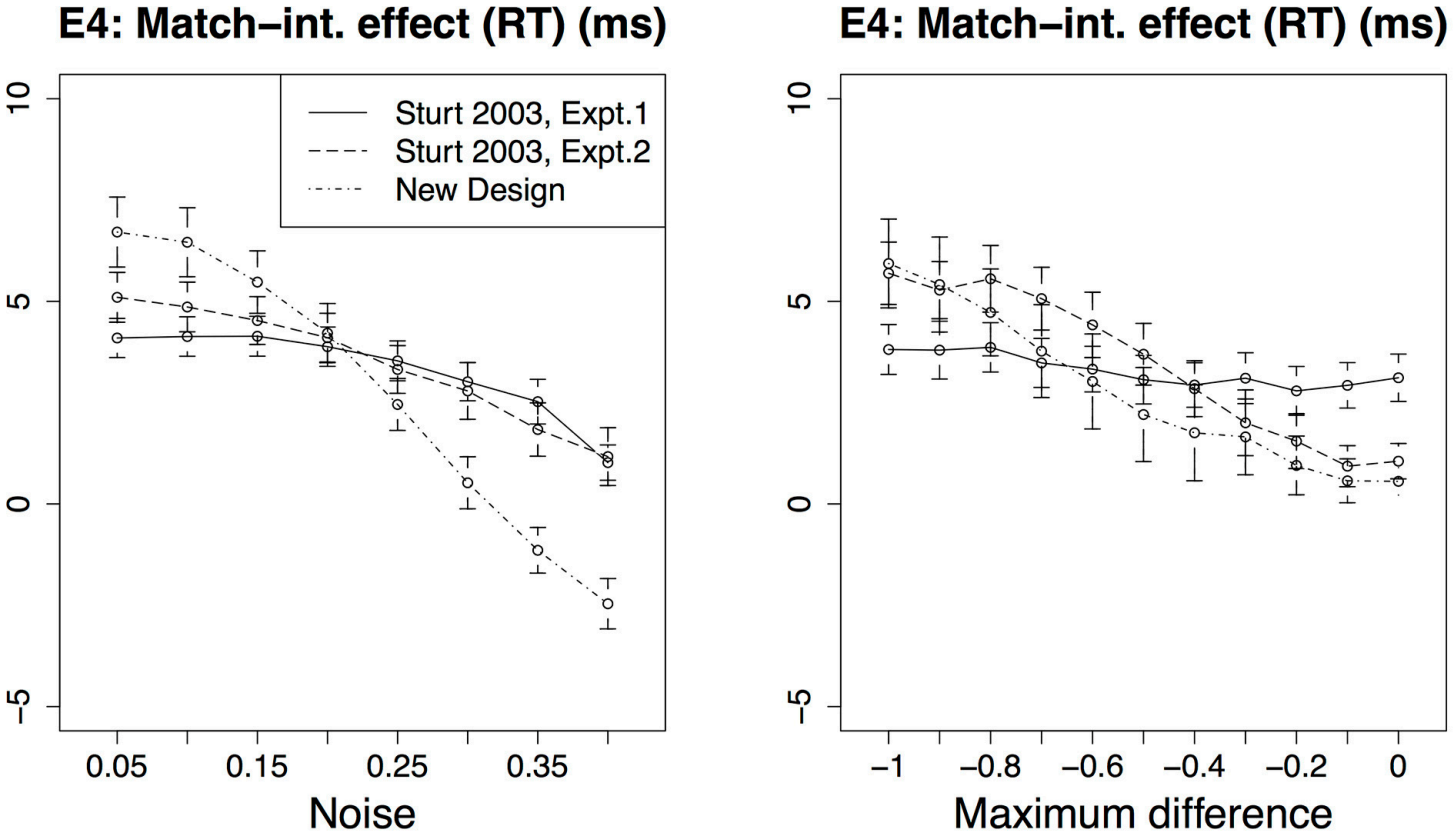
**The variations in effect E4 (match-interference (RT)) across noise and maximum difference parameters for three experimental designs**.

Figure 4 compares the predictions for effects E1–E5 across the three experimental designs. The predictions are generated for the range of parameter values listed in Table 2. The pattern of effects E1–E5 for Experiment 2 and the modified design are similar to that for Experiment 1. Across a range of parameter values, the predictions for effects E1, E2, and E5 are clearly stronger (higher numerical value) for the modified design than for Experiment 1 and 2 in Sturt (2003). Although the predictions for effect E3 are almost identical for the modified design and Experiment 2, they are nevertheless stronger than for Experiment 1. In contrast, the predictions for effect E4 are not distinguishable across the three designs. To gain better insight into the predictions for E4, we compared effect E4 across variations of the other two parameters (noise and maximum difference); see Figure 5. For the maximum difference parameter, effect E4 is stronger in the modified design and Experiment 2 than in Experiment 1 when the difference penalty is high (more negative), and it is weaker when the maximum difference penalty is low. For the noise parameter, effect E4 is stronger in the modified design and Experiment 2 than in Experiment 1 when the noise is low, and it is weaker when the noise is high. These patterns show that the predicted strength of effect E4 is dependent on the specific value or a range of values that are selected for these parameters. The noise parameter is a frequently modified parameter across various models (Wong et al., 2010), which is suggestive of uncertainty regarding its value across diverse cognitive tasks (cf. the decay parameter, which is usually kept fixed). The best way to estimate or, at least, restrict the noise parameter's value would be to empirically validate predictions of various models. In contrast to noise, the maximum difference penalty parameter is seldom modified, and is set to its default value of −1. For the default value of this parameter, the model clearly predicts a stronger E4 effect for the modified design and Experiment 2.

In sum, the predictions for the modified design and Experiment 2 show stronger effects than Experiment 1 across a range of parameter values. For the most part—and as expected—the effects for the modified design are much stronger than the other two designs. Next, we report an eye tracking study that we ran with the modified design (9). The goal of this study is to evaluate the predictions of our model that diverge from Sturt's findings (specifically, effects E4 and E5), as well as to replicate the effects E1–E3 that Sturt (2003) found.

## 3. Eye tracking experiment

### 3.1. Participants

Forty English native speakers residing in Berlin, Germany participated in the eye tracking study. Data from one participant was excluded due to less than 40% accuracy on the sentence comprehension questions on all trials including experimental and filler trials. The remaining 39 participants included 20 female participants and had a mean age of 29.5 years. The 39 native English speakers consisted of 14 British, 13 American, 8 Australian, 3 Canadian, and 1 New Zealander. All participants had normal or corrected-to-normal vision and were paid 10 Euros for their participation. The experiment had a duration of approximately 45 min, including set-up time. This study was carried out in accordance with the Helsinki declaration with written informed consent from all participants.

### 3.2. Design and materials

Twenty-four stimuli were selected from the Xiang et al. (2009) study and constructed as per (9) by adding an extra *match* condition (see Appendix B in Supplementary Material for the list of stimuli). Of these 24 stimuli, 12 used stereotypically male nouns and 12 used stereotypical female nouns for the binding-accessible antecedent. There were 4 lists that comprised different item-condition combinations according to a Latin Square. Each list contained 54 filler sentences. Two-third of the target items and all fillers contained a comprehension question, and these were equally distributed across yes and no answers. In all, each participant answered 70 comprehension questions.

### 3.3. Procedure

Participants were seated 60 cm from an NEC Multisync 2080UX screen color monitor with 1600 × 1200 pixel resolution. They were asked to sit comfortably in front of an EyeLink 1000 eye tracker (SR Research) running at 500 Hz sampling rate (0.01° tracking resolution, and < 0.5° gaze position accuracy). Though the viewing was binocular, only the participant's right eye was tracked. The distance between the camera and the eye was 50 cm.

Participants were asked to position their head in a frame that stabilized their forehead and chin. They were asked to avoid large head movements throughout the experiment and to avoid blinking while reading the sentences. A 7-button Microsoft Sidewinder game pad was used to record button responses. The presentation of the materials and the recording of responses was controlled by two separate PCs, one running internally developed software (this is called EyeScript, and was originally developed in Richard Lewis' lab by Mason Smith, and later in Shravan Vasishth's lab by Felix Engelmann, Titus von der Malsburg, and Tobias Günther; the software is open source and available at https://github.com/tmalsburg/EyeScript) and the other running SR Research's proprietary software.

Each participant was randomly assigned one of four different stimulus lists. The list was randomized for every subject. At the start of the experiment, a standard calibration procedure was performed which involved participants looking at a grid of 13 fixation targets in random succession, in order to validate their gazes. Calibration and validation were repeated if the experimenter noticed that measurement accuracy was poor, and if participants took a break during the experiment.

Each trial consisted of the following steps: First, a fixation target in the same position as the first character of the text display was presented; two 200 ms fixations followed by one 400 ms fixation on this target triggered the presentation of the sentences (this procedure ensured that the participants always started reading in the left-most character position and helped the experimenter ensure the accuracy of calibration). Participants were instructed to read the sentence at a normal pace and to move their gaze to a dot at the bottom right of the screen after finishing the sentence. This triggered the presentation of a comprehension question in two-thirds of the trials, and in the rest it triggered the presentation of the next trial. The comprehension questions were included in order to ensure that the participants attended to the content of the sentences.

### 3.4. Data analysis

All data processing and analyses were carried out in GNU-R (R Development Core Team, 2009). Fixations were detected using the algorithm described by Engbert and Kliegl (2003); an open source R package, *saccades*, developed by Titus von der Malsburg was used to carry out this step (the package is available at https://github.com/tmalsburg/saccades). Fixation and regression-based measures were extracted using another open source R package, *em2*, developed by Pavel Logačev (the package is available at: https://cran.r-project.org/src/contrib/Archive/em2/). All fixations 30 pixels above and below the sentence were included in the sentence. Fixations in the blank spaces between words were also counted; fixations in the first half of the space were included in the fixations on the preceding word and fixations in the second half were included in the fixations on the following word. All other fixations outside these regions were excluded.

Effects of accessible antecedent gender match (henceforth, *match*) and inaccessible antecedent gender match (*interference*) with the gender of the reflexive were evaluated across various eye movement measures. Data analysis was carried out using linear mixed models (Bates and Sarkar, 2007; Gelman and Hill, 2007). Linear mixed models were fit for the following eye movement measures at the reflexive. *First Fixation Duration* (FFD), the time spent during the first fixation during the first pass; *First Pass Reading time* (FPRT), the sum of all fixations during the first pass; *Re-reading Time* (RRT), the sum of all fixations in a region that occurred after first pass; *Total Reading Time* (TRT), the sum of all fixations in a region, *First Pass Regression Probability* (FPRP), the probability of regressing from a region after fixating in that region during first pass; and Re-reading Probability (RRP), the probability of reading a region during the second and subsequent passes. In the linear models, we used nested contrast coding and defined three contrasts that correspond to the three effects that we are interested in—mismatch effect, match-interference effect, and mismatch-interference effect. The interference effects were nested within the mismatch effect. The contrasts were coded such that having a positive coefficient meant that the effect was in the predicted direction. Apart from these three contrasts, trial number was used as a (centered) predictor. All linear mixed models were fit with by-participant and by-item random intercepts, and by-participant and by-item random slopes for the three contrasts. For FPRP and RRP, only random intercepts were used, since otherwise the models failed to converge. All reading times were log transformed before fitting the linear models. For FPRP and RRP, generalized linear mixed models were fit with a binomial link function.

### 3.5. Results

All mean reading times at the reflexive along with standard errors, FPRP from the reflexive, RRP at the reflexive and comprehension accuracy percentages are summarized in Table 3. The results of the statistical analysis are summarized in Tables 4, 5.

**Table 3:** **Mean reading times at the reflexive with standard errors, percentages of first pass regressions from the reflexive, percentages of re-readings of the reflexive, and comprehension question response accuracies across four conditions**.

**Condition**	**FFD**	**FPRT**	**RRT**	**TRT**	**FPRP (%)**	**RRP (%)**	**Accuracy (%)**
a. Match-interf.	258 (6)	280 (8)	108 (14)	410 (18)	13	30	84
b. Match	263 (7)	292 (10)	79 (11)	389 (17)	7	24	90
c. Mismatch-interf.	272 (8)	295 (10)	149 (20)	473 (24)	11	35	83
d. Mismatch	266 (9)	284 (9)	145 (15)	468 (20)	11	42	80

**Table 4:** **Linear mixed-effects model estimates, standard errors and *t*-values across reading time measures; the asterisk indicates statistically significant (α = 0.05) effects**.

	**Effect**	**Estimate**	**Std. Error**	***t*-value**
FFD	Intercept	5.512	0.027	201.387
	Mismatch	0.023	0.026	0.89
	Match-interference	−0.022	0.034	−0.650
	Mismatch-interference	−0.025	0.038	−0.653
	Trial	0.001	0.001	1.751
FPRT	Intercept	5.575	0.029	194.555
	Mismatch	0.011	0.03	0.356
	Match-interference	−0.033	0.053	−0.626
	Mismatch-interference	−0.028	0.04	−0.718
	Trial	0.001	0.001	2.203^*^
RRT	Intercept	5.679	0.052	109.464
	Mismatch	0.043	0.066	0.65
	Match-interference	0.022	0.094	0.231
	Mismatch-interference	−0.067	0.082	−0.818
	Trial	−0.001	0.001	−0.748
TRT	Intercept	5.9	0.049	121.125
	Mismatch	0.146	0.043	3.41^*^
	Match-interference	0.036	0.057	0.637
	Mismatch-interference	0.016	0.057	0.273
	Trial	0	0.001	0.4

**Table 5:** **Linear model estimates, standard errors and *p*-values for FPRP and RRP; the asterisk indicates statistically significant (α = 0.05) effects**.

	**Effect**	**Estimate**	**Std. Error**	***p*-value**
FPRP	Intercept	−2.602	0.258	<10^−15^
	Mismatch	0.148	0.256	0.564
	Match-interference	0.782	0.374	0.037^*^
	Mismatch-interference	−0.111	0.35	0.751
	Trial	0.004	0.006	0.431
RRP	Intercept	−0.852	0.176	<10*^−^*^5^
	Mismatch	0.63	0.154	<10*^−^*^4^^*^
	Match-interference	0.359	0.225	0.11
	Mismatch-interference	0.383	0.206	0.063
	Trial	−0.006	0.003	0.067

#### 3.5.1. Question-response accuracy

Overall average accuracy for trials that included a comprehension question was 88% and average accuracy for target items was 84%. Accuracy values for comprehension questions across four conditions are listed in Table 3, but are not theoretically interpretable because the questions targeted different parts of the critical sentence, not just the antecedent-reflexive relation as in the Sturt follow-up study. We present these mean accuracies only for completeness.

#### 3.5.2. Eye tracking dependent measures

A statistically significant mismatch effect was observed in TRT and RRP, i.e., the conditions in which the stereotypical gender of the accessible antecedent did not match the gender of the reflexive were read more slowly and had higher probability of re-reading than the conditions where it matched. A statistically significant match-interference effect was observed in FPRP, with the high interference condition showing more regressions than the low interference condition.

### 3.6. Discussion

#### 3.6.1. Early effects

**Figure 6:**
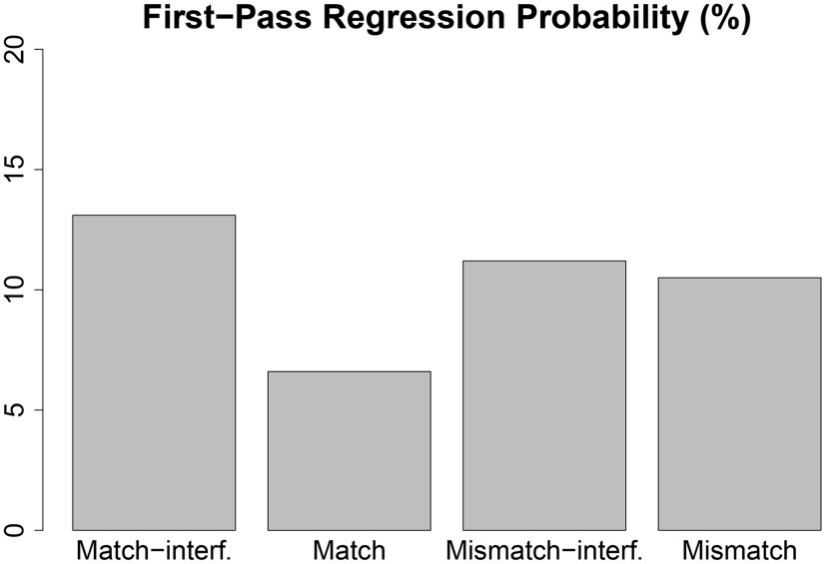
**Early effect of *interference***. Effects that are observed in “early” eye tracking measures are considered to be early effects. Usually measures like FFD, FPRT, and FPRP are considered to be early measures since they are associated with reader's first exposure to a region.

The results outlined above show an early effect of match-interference (E2) from the inaccessible antecedent in first pass regression probability, such that a gender match between the reflexive and the inaccessible antecedent leads to a higher number of first pass regressions from the reflexive (see Figure 6). The occurrence of a regression from a word reflects some difficulty in integrating the word when it is fixated and hence it is plausibly an early effect (Clifton et al., 2007). We are assuming that higher number of retrieval errors should reflect in higher probability of regression. First pass regressions cannot reflect the late processes triggered at the end of a sentence or the processes reflected by late measures such as second pass reading time. Assuming that first pass regressions reflect processing difficulty triggered relatively early during the first contact with the critical word, the interference effect is inconsistent with the conclusion of Sturt (2003), that the online application of Principle A is not affected by interference from the inaccessible antecedent at early stages of processing. Conclusions derived in Nicol and Swinney (1989) and Xiang et al. (2009) are also not compatible with these results. As a result, this study challenges the claim from Phillips et al. (2011) and Dillon et al. (2013) that an antecedent for a reflexive is retrieved using only structural cues without considering the gender feature. Our findings are consistent with those of Badecker and Straub (2002), Choy and Thompson (2010), Cunnings and Felser (2013), Thompson and Choy (2009).

#### 3.6.2. Late effects

**Figure 7:**
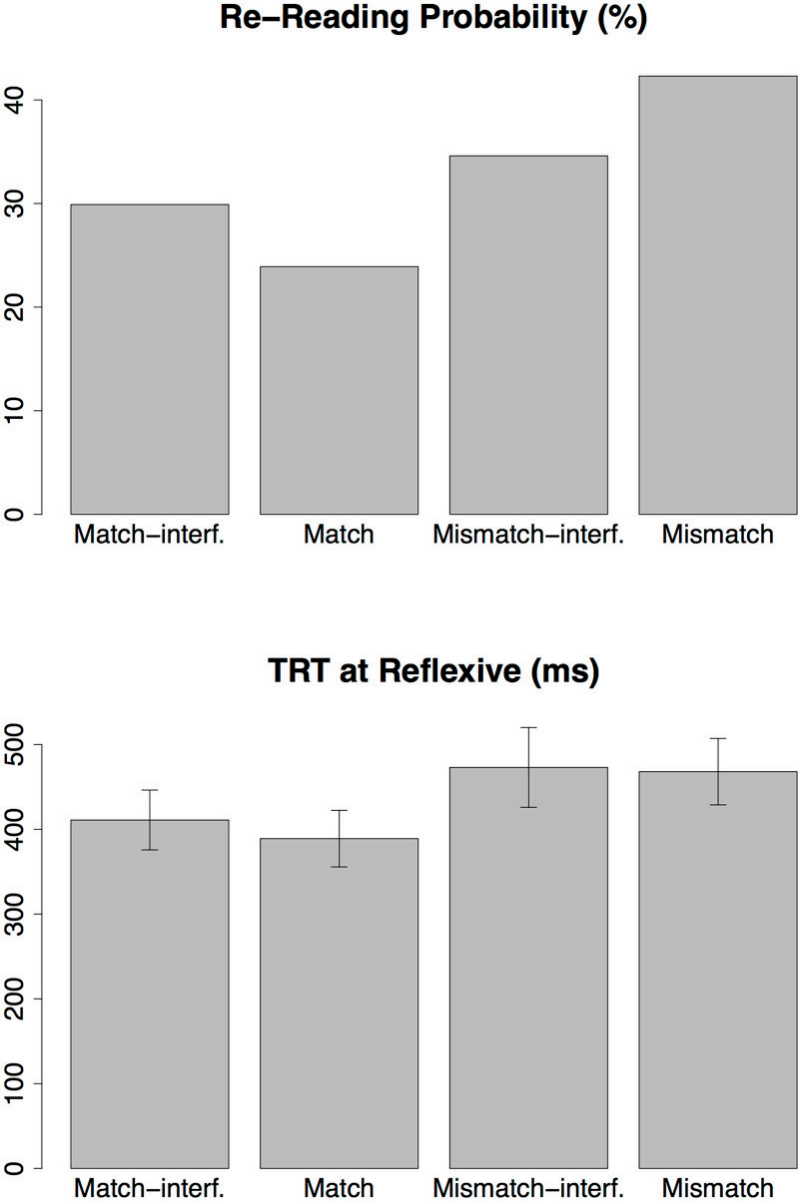
**Late effect of *match***. Effects that are observed in “late” eye tracking measures are considered to be late effects. Usually measures like RRP and TRT are considered to be late measures since they involve reader's second and possibly subsequent exposures to a region.

The effect of accessible antecedent gender match (E1 and E3) was also observed in the RRP and TRT (see Figure 7) such that reading times were elevated and there were higher number of re-readings when the accessible antecedent did not match the gender of the reflexive (we are assuming that E1, the mismatch effect predicted in retrieval errors, should reflect in elevated reading times). The absence of an early effect of accessible antecedent is different from the finding of Sturt (2003), where the effect appeared at FFD. We also observed a marginal effect of mismatch-interference (E5) (*p* = 0.063) in the RRP. Although the effect doesn't reach conventional significance level, it corroborates the patterns we observe in our exploratory data analysis with cumulative progressions (see Section 3.6.4).

#### 3.6.3. Regression contingent effects in FFD

**Figure 8:**
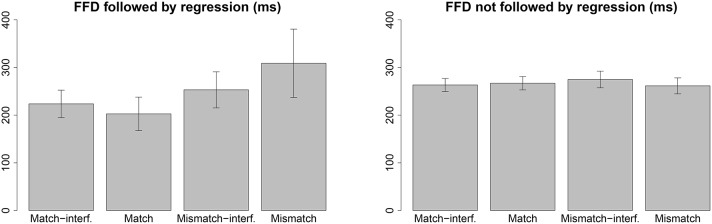
**Regression contingent FFD**.

As an exploratory data analysis, we analyzed FFD contingent on the first pass regressions—separate analysis for FFD followed by regressions and FFD not followed by regressions. The two patterns are plotted in Figure 8. FFD followed by regressions show a pattern consistent with the retrieval times predicted by the model. Although the match-interference effect (E4) (*t* = 1.77) and mismatch-interference effect (E5) (*t* = 1.70) do not quite reach conventional significance levels, they show the trend of interference effect as predicted by the model. These FFDs also show the main effect of mismatch (E1 and E3) (*t* = 2.78) which is consistent with the early mismatch effect in Sturt (2003). FFD not followed by regressions did not show this effect.

#### 3.6.4. Effects revealed in cumulative progressions

As another way of exploratory data analysis, we examined an eye movement measure called the cumulative progression, which has been used earlier by Kreiner et al. (2008) and Cunnings and Sturt (2014). The cumulative progression quantifies how far a reader's eyes have traveled from the region of interest. The assumption with analyzing cumulative progressions is that the further away, in the direction of reading, a reader progresses from a region (in one condition compared to another), the easier the information in that region is for processing. It is a measure of continuous eye movements, in the sense that it assigns a numeric value, the distance, at each point in time that can be recorded by an eye tracker. This makes it possible to compare the processing cost between two conditions over a continuous period of time. For example, in our case, we could examine if participants consistently progress further away from the reflexive region in the match conditions compared to the mismatch conditions after entering the reflexive region for the first time. And if they do, then, by assumption, it implies that the reflexives are easier to process in the match conditions than in the mismatch conditions. Effectively, we are assuming that faster retrievals at the reflexive will result (in faster processing, and hence) in progressions that are further away from the reflexive.

Cumulative progressions are computed by measuring the distance between the position of the first fixation in the region of interest and the subsequent eye positions, ignoring word boundaries. In the earlier studies mentioned above the distance was calculated in terms of characters (the number of characters by which the current eye position is separated from the position of the first fixation in the region of interest). Only forward eye movements change the value of the measure; regressive eye movements or no eye movements, as in fixations, do not change the value of the measure. This means that the sequence of cumulative progressions for one trial is a monotonically increasing sequence—every subsequent number (representing the distance) is greater than or equal to the previous number (hence the name cumulative). Unlike in earlier studies, where the distance was calculated in terms of characters, we calculate the distance in terms of the number of screen pixels a participant has progressed, which gives a more fine-grained measure of distance. As in Cunnings and Sturt (2014), we evaluate various effects by comparing the numerical *differences* in mean cumulative progressions for different conditions.

**Figure 9:**
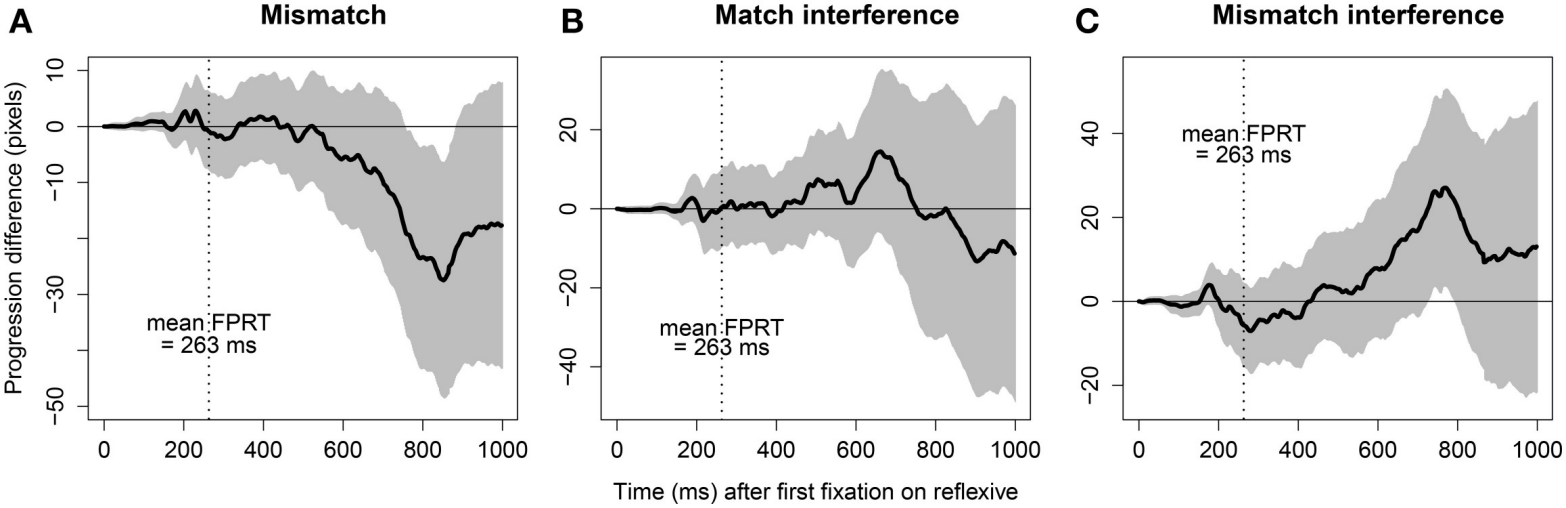
**Progression differences plotted against timestamps starting at the first fixation in the reflexive region**. Each curve represents an effect and the gray area around it represents the 95% confidence interval. The dotted vertical line denotes the position of the mean FPRT for the reflexives. **(A)** The mismatch effect is obtained by subtracting the mean progressions in the two mismatch conditions from the mean progressions in the two match conditions, **(B)** the match-interference effect is obtained by subtracting the mean progressions in the match condition from the mean progressions in the match-interference condition, and **(C)** the mismatch-interference effect is obtained by subtracting the mean progressions in the mismatch condition from the mean progressions in the mismatch-interference condition.

Figure 9 plots cumulative progression differences. Each panel represents one of the three effects in reading times that we are considering here. Each point on curve is obtained by first averaging cumulative progressions across participants and items for one condition at one timestamp, and then calculating the difference between the averages across two conditions that are compared. For the mismatch effect curve, the averaging is done for two pairs of conditions and then the difference between them is calculated. The x-axis represents timestamps starting with the first fixation in the reflexive region and extends till the next 1000 ms; two consecutive timestamps are 2 ms apart since the eye tracker sampled every 2 ms (which means each curve is composed of 501 points). The y-axis represents the difference in pixels between averaged progressions of conditions that are compared. It is crucial to note here that this is an analytic approach, and it is only an exploratory data analysis. Since each data point in this figure is averaged across participants and items, it underestimates variance between participants and items. Moreover, the 95% confidence intervals are underestimates, and with more conservative approach the plots may look consistent with noise. Overall, we need a more rigorous statistical analysis to do justice to the conclusions we are drawing based on cumulative progressions.

The curve representing the mismatch-interference effect diverges at 434 ms from the x-axis on the positive side and remains on the positive side. This implies that after the first fixation in the reflexive region, from 434 ms onwards, participants speed up in the mismatch-interference condition compared to the mismatch condition. This, in turn, is consistent with the mismatch-interference effect (E5) predicted by the model. The curve representing the mismatch effect diverges at 528 ms from the x-axis on the negative side and remains on the negative side. This implies that the two mismatch conditions are read slower than the two match conditions from 528 ms onwards, after the first fixation in the reflexive region, which is consistent with the mismatch effect (E3) predicted by the model. However, the match-interference effect curve diverges from the x-axis, initially on the positive side at 420 ms and then switches to the negative side at 754 ms and then predominantly remains on the negative side. The diversion in the positive direction is opposite to what the model predicts, but the later diversion to the negative side is consistent with the predictions of the model. Effectively, if we assume that faster cumulative progressions from the reflexive region reflect faster retrievals at the reflexive, the mismatch-interference effect (E5) and the mismatch effect (E3) are visible in the cumulative progressions. Interestingly, the mismatch-interference effect also starts earlier than the mismatch effect.

#### 3.6.5. Timing of mismatch and interference effects

It is important to note that the predictions of the model are not specific to early or late measures, but we expect that both mismatch and interference effects should occur during the same time frame in an experiment because, in the model, both the effects take place during the same (sub)process. Moreover, the absence of early mismatch effect in our experiment (and also in Cunnings and Felser, 2013 and Cunnings and Sturt, 2014) does not support the argument in Sturt (2003) that binding accessible antecedents influence early stages of processing, albeit it need not necessarily speak *against* Sturt's argument either, because early effects don't always show up in early measures (Vasishth et al., 2013).

In sum, the eye tracking study, through various measures, supported the predictions of the cue-based retrieval model of reflexive binding that assumes gender of the reflexive as one of the retrieval cues. The mismatch effects E1 and E3 were observed in total reading time, re-reading probability and in cumulative progressions from the reflexive. The interference effect E2 was observed in first pass regression probability. The interference effect E4 was observed in first pass regression probability and a trend of this effect was observed in the regression contingent first fixation duration. The interference effect E5 was observed in re-reading probability, cumulative progressions from the reflexive, and there was also a trend of this effect in first fixation durations that were followed by regressions. Along with replicating the mismatch effects, observed in Sturt's experiments, the presence of interference effects (E4 and E5), which were absent in Sturt's experiments, makes the results consistent with the model's predictions.

## 4. General discussion

In this paper, we investigated the question: what kinds of cues are used initially by the parser when resolving antecedent-reflexive relations? The two positions on this question are: early use of only structural cues (Nicol and Swinney, 1989; Sturt, 2003; Xiang et al., 2009; Phillips et al., 2011; Dillon et al., 2013), or early use of structural as well as other cues such as gender marking (Badecker and Straub, 2002). We framed the theoretical question within a computational model of sentence processing, the cue-based retrieval model proposed in Lewis and Vasishth (2005) and Lewis et al. (2006), and showed that if we assume that cue-based retrieval involves structural as well as non-structural cues, the model makes five predictions, repeated below:

E1. Mismatch effect (RE): the retrieval errors for the two mismatch conditions are higher than those for the two match conditions.
E2. Interference effect (RE): the retrieval errors for the match-interference and mismatch-interference conditions are higher than those for the other two conditions.
E3. Mismatch effect (RT): the retrieval times for the two mismatch conditions are longer than those for the two match conditions.
E4. Match-interference effect (RT): the retrieval times for the match-interference condition are longer than the match condition.
E5. Mismatch-interference effect (RT): the retrieval times for the mismatch-interference condition are *shorter* than the mismatch condition.

Effects E1–E3 are attested in Sturt's studies; but effects E4 and E5 are not. We hypothesized that Sturt failed to find effect E4 because the inaccessible antecedent had a different grammatical role (object) than the accessible antecedent (subject); i.e., it was distinct enough from the accessible antecedent to be rejected successfully during search. We predicted that if both the accessible and inaccessible antecedents had the subject role, then a match-interference (E4) effect would occur. Moreover if grammatical cues are weighted heavily in the retrieval process (Van Dyke and McElree, 2011), a subject distractor will induce a higher interference effect. We then conducted an eye tracking study in which both the accessible and inaccessible antecedents had the subject role, thereby increasing their similarity. We showed that in first pass regression probability a match-interference effect is indeed seen, as predicted by the model. In addition, as an exploratory data analysis, when we separately analyzed the first fixation duration contingent on regressions, the first fixation durations that were followed by regressions showed marginal effects consistent with the two interference effects E4 and E5. These first fixation durations also confirmed the effect E3. The effect E3 was observed in re-reading time and total reading time as well. This result is consistent with the model's predicted mismatch effect in retrieval times, and the predicted mismatch effect in retrieval errors. Further, in another exploratory data analysis with an eye tracking measure called cumulative progressions, which has been claimed to capture processing difficulty on a continuous time scale, we found that the interference effect E5 and the mismatch effect E3 are realized; with E5, in fact, occurring earlier than E3. Though the analysis with cumulative progressions involved only visual inspection, the visual patterns are consistent with these two effects predicted by the model.

In sum, the eye tracking study provided empirical evidence for all the effects predicted by the model, including the interference effects that were not observed in the earlier studies such as Sturt (2003) and Xiang et al. (2009). There was clear support for the mismatch and match-interference effect predicted by the model. Although the support for the mismatch-interference effect was not equally clear—it was only marginally significant in two of the eye tracking measures, and there was some evidence in the exploratory data analysis with cumulative progressions—the two interference effects have important theoretical implications for the generality of the retrieval mechanisms in sentence processing, and so should not be ignored.

The interference effects and the mismatch effect have also been observed in some other studies^5^. The mismatch effect E3 has been found in the reading studies (eye tracking and/or self-paced reading) such as Cunnings and Felser (2013) (Experiment 1 and 2), Cunnings and Sturt (2014) (Experiment 1), Dillon et al. (2013) (Experiment 1 and 2), King et al. (2012), Parker and Phillips (2014) (Experiment 1, 2, and 3), and Sturt and Kwon (2013) (Experiment 3 and 4) with a design comparable to Sturt's experiment 1. The interference effect E4 has been found in the reading studies such as Badecker and Straub (2002) (Experiment 3 and 4) and Mansbridge and Witzel (2012), and the interference effect E5 has been found in the reading studies such as Cunnings and Felser (2013) (Experiment 2 in high working memory span readers), King et al. (2012), Parker and Phillips (2014) (Experiment 2 and 3) Sturt and Kwon (2013) (Experiment 3 and 4). In the visual world paradigm, an effect equivalent to the interference effect E4 has been reported in Choy and Thompson (2010), Clackson et al. (2011), Runner and Head (2014), and Thompson and Choy (2009). Overall the pattern appears to be that the mismatch effect has been observed robustly, although there are at least a handful of studies reporting the two interference effects as well.

### 4.1. Why were the interference effects found less often in earlier studies?

Apart from the reasons mentioned in the motivation for the design of the experiment reported here, namely the proximity of the inaccessible antecedent to the reflexive and it being the subject of the clause, there could be other reasons for the absence of the interference effect. The absence of the effect could just be a failure to find an effect that in fact exists, which may happen due to low power of the experiment. For example, the effect could be masked by other confounding variables. Indeed, Cunnings and Felser (2013, p. 23) found that participants with high working memory spans show (in first fixation duration) an effect in exactly the direction predicted by the cue-based retrieval model (though they didn't interpret the effect as an interference or intrusion effect). It is participants with low working memory span who show longer first fixation durations in the interference condition in the mismatch cases. If one were to ignore the working memory span in the Cunnings and Felser data, the two differently-signed effects by span would cancel out, showing no difference between the interference and no-interference condition in the mismatch cases, exactly as found in the literature. Thus, since our data and all previous experiments (except, of course, Cunnings and Felser's) do not take working memory capacity into account as a variable, it is quite possible that we are missing an effect that is correctly predicted by the model. Of course, this raises the question that the ACT-R model as currently implemented does not explicitly model high working memory capacity participants. In future work, we intend to explore the role of working memory capacity in triggering the mismatch interference effect.

Another possibility could be that the interference effects are not as strong as the mismatch effect. The model, in fact, predicts numerically smaller interference effects compared to the mismatch effect (see Figure 4). Recently, Parker and Phillips (2014) using sentences as in (10), found that the mismatch-interference effect is visible when the reflexive mismatches two features, such as the number and gender (e.g., herself and schoolboys), with the accessible antecedent, but not when it mismatches only one feature. King et al. (2012) using sentences as in (11), found that the mismatch-interference effect is visible when the reflexive is not adjacent to the verb [condition (b) in (11)] allowing the information about the verb's argument structure, and hence the information about the accessible antecedent, to decay. These results possibly corroborate the model's prediction that the mismatch-interference effect is weaker than the mismatch effect, and hence difficult to detect.

(10) The {librarian/janitor} said that the {schoolgirl/schoolboy/schoolboys} reminded herself about the book.
(11)
  a. *Verb-adjacent:* The mechanic who spoke to {John/Mary} sent {himself/herself} a package.
  b. *Verb-non-adjacent:* The mechanic who spoke to {John/Mary} sent a package to {himself/herself}.

Effectively, our results are not only in line with the results from Cunnings and Felser (2013), King et al. (2012), and Parker and Phillips (2014), among others, but also provide convincing evidence for the model's predictions with manipulations independent of the memory span of the participants, with a configuration involving verb-adjacent reflexives, and with lowest possible (=single) mismatch of retrieval cues with the accessible antecedent.

#### 4.1.1. Strictly structured access as an alternative

Here, we discuss the strictly structured retrieval approach proposed in Dillon et al. (2013) and Phillips et al. (2011) for resolving reflexive-antecedent dependency, and examine its claims in the light of existing experimental and modeling findings. Although Phillips and colleagues refers to the mechanism as structured access, we refer to it as a *strictly* structured access to emphasize the point that the approach suggested in this paper does not ignore the structural constraints, but it includes other constraints as well.

Dillon et al. (2013) supported evidence for the strictly structured access with a set of computational and experimental studies involving English reflexives and subject-verb agreement. This experiment essentially replicated the *interference asymmetry* effect from Wagers et al. (2009) and the absence of interference effect in processing English reflexives from Sturt (2003). Based on these results, they concluded that agreement dependency and reflexive dependency employ different retrieval mechanisms for resolving the dependencies—agreement dependencies are resolved using morphological features of the target noun phrase whereas the antecedent for a reflexive is retrieved using *only* structural constraints. They further compared the predictions of a strictly structural cue based retrieval model of reflexives to a model utilizing mixed cues—structural as well as agreement. The mixed cue model predicted an interference effect in retrieval errors (similar to E2 above) and a mismatch-interference effect in retrieval times (similar to E5 above). The prediction of the match-interference effect (E4) was not reliably non-zero, in the sense that for some parameter combinations the model didn't predict any difference between the match and match-interference condition. The structural cue based model predicted no interference effect in either retrieval errors or retrieval times. The mismatch effects in retrieval errors and retrieval times (E1 and E3), as predicted by the mixed cue model, were not discussed in Dillon et al. (2013). On the basis of these predictions, Dillon et al. (2013) concluded that the strictly structured access model captures the reflexive binding data from their Experiments better than the mixed cue model.

Although Dillon et al. (2013) replicated the findings in Sturt (2003), the lack of interference effect is subject to the same alternative explanation that we suggested for Experiment 2 in Sturt (2003): we hypothesized that reflexive binding uses the grammatical role *subject* as one of the retrieval cues for retrieving the correct antecedent. The absence of the interference effect could be due to (apart from power concerns) the fact that the interfering antecedent had an *object* role in the experiments above, which does not match one of the retrieval cues, reducing the strength of interference. Badecker and Straub (2002) also reported that the interference effect is found when the interferer is in the subject position. Moreover, Van Dyke and McElree (2011) found that, in thematic binding, the interference effect due to the semantic match was present only when the distractors matched syntactic cues along with semantic cues. If the retrieval process gives higher weight to syntactic cues than semantic cues, the absence of interference effect could simply be due to the absence of matching a grammatical role in the inaccessible antecedent.

**Figure 10:**
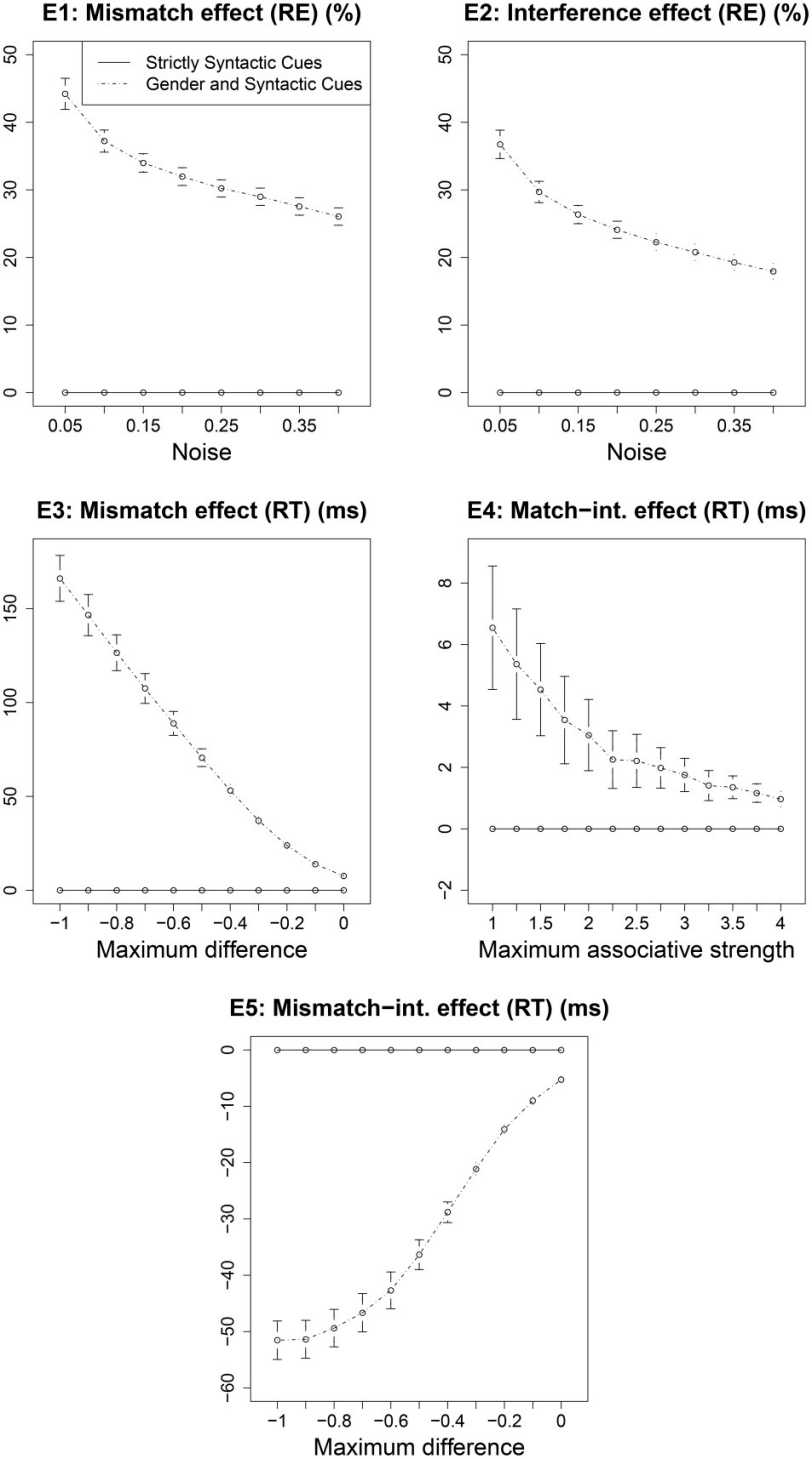
**The predictions of a strictly structural cue-based model and a model with structural and agreement feature based cues**. The predictions are for the new experimental design and are generated across a set of parameter values.

The predictions of the structured access model hold only for a limited set of experiments and a limited set of effects in those experiments. The mismatch-effects (E1 and E3) that have been replicated in various studies like Sturt (2003), Cunnings and Felser (2013) and also the one reported in this paper cannot be explained by this model. The structured access model predicts no difference between match and mismatch conditions (see Figure 10). Furthermore, the interference-effects (E2, E4, and E5) observed in various reflexives studies like Badecker and Straub (2002) Experiment 3, Sturt (2003) follow-up study, Cunnings and Felser (2013) Experiment 2 and the one reported here cannot be explained by this model. Consequently, a model assuming structural as well as agreement features as retrieval cues predicts a broader range of data than a strictly structured access model.

Dillon et al. (2013) further claimed that the match-interference effect (E4)—higher reading times when the inaccessible noun matches gender or number of the reflexive—is not reliable evidence for interference from the grammatically inaccessible antecedent, for mainly two reasons: (1) The cue-based retrieval model doesn't predict any difference in retrieval times between the match and match-interference conditions for certain parameter combinations, since on the one hand the cue-overlap (gender and number) between accessible and inaccessible antecedents leads to an inhibitory effect, and on the other hand the retrieval of the inaccessible antecedent leads to a facilitatory effect; (2) The match-interference effects can be explained in terms of feature-overwriting (Nairne, 1990; Gordon et al., 2001, 2004, 2006; Oberauer and Kliegl, 2006) instead of interference at the time of retrieval. Consequently, Dillon proposed that only a facilitatory effect in mismatch-interference can be considered as evidence for retrieval interference.

As far as the first argument is concerned, we, in fact, show that the cue-based retrieval model with mixed cues consistently predicts a positive match-interference effect across a set of parameter values for Sturt's two experiments (see Figures 4, 5). Although the effect for the modified design is not predicted to be positive for all combinations of parameter values, for a certain set of combination of values the effect is non-zero and positive, and only for a very small set of parameter values is the effect predicted to be zero.

The second argument, in fact, applies to the mismatch-interference effect as well—the mismatch-interference effect (for this particular design) can also be explained in terms feature-overwriting or encoding interference. Encoding interference is a consequence of the feature overlap between the accessible and inaccessible antecedents. In the design discussed here, the feature overlap between the accessible and inaccessible antecedents is, in fact, higher in the mismatch condition compared to the mismatch-interference condition (“soldier” and “Fred” have the same gender in the mismatch condition whereas “soldier” and “Katie” have different genders in the mismatch-interference condition). This means between these two conditions, the interference conditions are reversed for encoding and retrieval interference. Retrieval interference predicts faster reading time for the mismatch-interference condition while encoding interference predicts *slower* reading time for the *mismatch* condition, leading to exactly the same pattern of retrieval times between the two mismatch conditions. Effectively, this configuration makes it impossible to tease apart the two types of interference theories using the experiment design considered in this paper or in similar earlier studies including Dillon et al. (2013). However, Jäger et al. (2015a) using self-paced reading and eye-tracking studies with German and Swedish reflexives, compared the predictions of the two interference theories. They could not find any evidence for encoding interference and concluded that “invoking encoding interference may not be a plausible way to reconcile interference effects with a structure-based account of reflexive processing.” If we assume that the retrieval process for reflexives in German, Swedish and English are similar (especially because these are closely related languages) then we can safely conclude that, even though our design does not have the possibility of disentangling the two retrieval theories, the effects that we see in our experiment are driven by retrieval interference.

In summary, we have presented a theory and computational model of the access of antecedents for reflexive pronouns in English, and used this theory to gain insight into empirical studies that have yielded mixed results concerning the putative role of non-structural cues. We used this analysis and the results of further modeling to motivate a new empirical design that formed the basis of an eye tracking study. Many of the results of the eye tracking study are consistent with the model's assumptions concerning the early use of non-structural cues. These results present a challenge for theories advocating the infallibility of the human parser in the case of reflexive binding in English, and provide support for the inclusion of agreement features such as gender in the set of retrieval cues. In general, the results provide further support for the deployment of a rapid, parallel cue-based access mechanism in service of sentence parsing (McElree, 2000; McElree et al., 2003; Lewis and Vasishth, 2005; Lewis et al., 2006), and help to sharpen deeper explanatory questions concerning the utility and selection of cues.

## Author contributions

UP wrote the model, SV and RL supervised the process. UP conceived, setup, and carried out the experiment. UP and SV analyzed the data. UP, SV, and RL wrote the manuscript.

### Conflict of interest statement

The authors declare that the research was conducted in the absence of any commercial or financial relationships that could be construed as a potential conflict of interest.

## Footnotes

^1^Principle A specifies a structural constraint on the interpretation of reflexives in English: a reflexive must be bound by an antecedent in the local domain (the current clause). An antecedent X can bind a reflexive Y, if X and Y are coindexed, and X c-commands Y. The term c-command defines a hierarchical relationship between two constituents in a syntax tree. A constituent c-commands its sister constituent and every constituent *below* the sister constituent in the syntax tree. In (1), the reflexive *himself* is bound by *surgeon*; the noun *Jonathan* cannot bind the reflexive because it does not c-command it.

^2^In this paper, we follow the literature (see e.g., Sturt, 2003) in assuming that so-called early and late measures in eye tracking data map onto processes that occur (respectively) in early and late stages of parsing.

^3^We will follow the literature in referring to the correct antecedent as stipulated by Principle A as the *grammatically accessible* antecedent and the antecedent that is incorrect following Principle A as the *grammatically inaccessible* antecedent. Occasionally, we abbreviate these terms to *accessible* and *inaccessible* antecedents. It is important to keep in mind that under the model we advocate in this paper, the grammatically inaccessible antecedent is in fact “accessible” for memory retrieval; a more appropriate term would have been “incorrect antecedent,” since this does not presuppose that the non-c-commanding antecedent is inaccessible.

^4^There are more sophisticated ways to encode the c-command constraint but these implementation details are orthogonal to the present discussion. See Alcocer and Phillips (2012) which compares some alternatives of implementing the c-command constraint.

^5^In this paper we are considering only argument reflexives and only in English. But interference effects have also been reported for Mandarin reflexives (Jäger et al., 2015b; Chen et al., 2016) and for non-argument positions such as English reflexives inside picture noun phrases (Runner et al., 2006). On the other hand, for the pronoun-antecedent dependency (which is subject to different grammatical constraints than the reflexive-antecedent dependency), Chow et al. (2014) failed to replicate the interference effect observed in the pronoun experiments reported in Badecker and Straub (2002).

## References

[B1] Aho A. V. Ullman J. D. (1972). The Theory of Parsing, Translation and Compiling Volume I: Parsing. Upper Saddle River, NJ: Prentice-Hall.

[B2] Alcocer P. Phillips C. (2012). Using Relational Syntactic Constraints in Content-Addressable Memory Architectures for Sentence Parsing. Unpublished manuscript. Available online at: http://www.colinphillips.net/wp-content/uploads/2014/08/alcocer_phillips2012_v2.pdf (Retrieved March 4, 2016).

[B3] Anderson J. R. Bothell D. Byrne M. D. Douglass S. Lebiere C. Qin Y. (2004). An integrated theory of the mind. Psychol. Rev. 111, 1036–1050. 10.1037/0033-295X.111.4.1036 15482072

[B4] Badecker W. Straub K. (2002). The processing role of structural constraints on the interpretation of pronouns and anaphors. J. Exp. Psychol. Learn. Mem. Cogn. 28, 748–769. 10.1037/0278-7393.28.4.748 12109766

[B5] Bates D. Sarkar D. (2007). lme4: Linear Mixed-Effects Models Using S4 Classes (R Package Version 0.9975-11).

[B6] Chen Z. Jäger L. Vasishth S. (2016). How structure sensitive is the parser? Evidence from Mandarin Chinese, in Empirical Approaches to Linguistic Theory: Studies of Meaning and Structure, Studies in Generative Grammar, eds Stolterfoht B. Featherston S. (Berlin: Mouton de Gruyter), 43–62.

[B7] Chomsky N. (1981). Lectures on Government and Binding. Berlin: Mouton de Gruyter.

[B8] Chomsky N. (1986). Barriers. Cambridge, MA: MIT Press.

[B9] Chow W. Y. Lewis S. Phillips C. (2014). Immediate sensitivity to structural constraints in pronoun resolution. Front. Psychol. 5:630. 10.3389/fpsyg.2014.00630 25018739 PMC4073625

[B10] Choy J. J. Thompson C. K. (2010). Binding in agrammatic aphasia: processing to comprehension. Aphasiology 24, 551–579. 10.1080/02687030802634025 20535243 PMC2882310

[B11] Clackson K. Felser C. Clahsen H. (2011). Children's processing of reflexives and pronouns in English: evidence from eye-movements during listening. J. Mem. Lang. 65, 128–144. 10.1016/j.jml.2011.04.007

[B12] Clifton C. Staub A. Rayner K. (2007). Eye movements in reading words and sentences, in Eye Movements: A Window on Mind and Brain, ed van Gompel R. (Amsterdam: Elsevier), 341–372.

[B13] Cunnings I. Felser C. (2013). The role of working memory in the processing of reflexives. Lang. Cogn. Process. 28, 188–219. 10.1080/01690965.2010.548391

[B14] Cunnings I. Sturt P. (2014). Coargumenthood and the processing of reflexives. J. Mem. Lang. 75, 117–139. 10.1016/j.jml.2014.05.006

[B15] Dillon B. Mishler A. Sloggett S. Phillips C. (2013). Contrasting intrusion profiles for agreement and anaphora: experimental and modeling evidence. J. Mem. Lang. 69, 85–103. 10.1016/j.jml.2013.04.003

[B16] Engbert R. Kliegl R. (2003). Microsaccades uncover the orientation of covert attention. Vision Res. 43, 1035–1045. 10.1016/S0042-6989(03)00084-1 12676246

[B17] Frazier L. (1979). On Comprehending Sentences: Syntactic Parsing Strategies. PhD thesis, University of Massachusetts, Amherst, MA.

[B18] Gelman A. Hill J. (2007). Data Analysis Using Regression and Multilevel/Hierarchical Models. New York, NY: Cambridge University Press.

[B19] Gordon P. C. Hendrick R. Johnson M. (2001). Memory interference during language processing. J. Exp. Psychol. Learn. Mem. Cogn. 27, 1411–1423. 10.1037/0278-7393.27.6.1411 11713876

[B20] Gordon P. C. Hendrick R. Johnson M. (2004). Effects of noun phrase type on sentence complexity. J. Mem. Lang. 51, 97–114. 10.1016/j.jml.2004.02.003

[B21] Gordon P. Hendrick R. Johnson M. Lee Y. (2006). Similarity-based interference during language comprehension: evidence from eye tracking during reading. J. Exp. Psychol. Learn. Mem. Cogn. 32, 1304–1321. 10.1037/0278-7393.32.6.1304 17087585 PMC1766329

[B22] Jäger L. A. Benz L. Roeser J. Dillon B. W. Vasishth S. (2015a). Teasing apart retrieval and encoding interference in the processing of anaphors. Front. Psychol. 6:506. 10.3389/fpsyg.2015.00506 26106337 PMC4460324

[B23] Jäger L. A. Engelmann F. Vasishth S. (2015b). Retrieval interference in reflexive processing: experimental evidence from Mandarin, and computational modeling. Front. Psychol. 6:617. 10.3389/fpsyg.2015.00617 26074829 PMC4444751

[B24] King J. Andrews C. Wagers M. (2012). Do reflexives always find a grammatical antecedent for themselves?, in The 25th Annual CUNY Conference on Human Sentence Processing, New York, NY.

[B25] Kreiner H. Sturt P. Garrod S. (2008). Processing definitional and stereotypical gender in reference resolution: evidence from eye-movements. J. Mem. Lang. 58, 239–261. 10.1016/j.jml.2007.09.003

[B26] Lewis R. L. Vasishth S. (2005). An activation-based model of sentence processing as skilled memory retrieval. Cogn. Sci. 29, 375–419. 10.1207/s15516709cog0000_25 21702779

[B27] Lewis R. L. Vasishth S. Van Dyke J. A. (2006). Computational principles of working memory in sentence comprehension. Trends Cogn. Sci. 10, 447–454. 10.1016/j.tics.2006.08.007 16949330 PMC2239011

[B28] Mansbridge M. P. Witzel J. (2012). Binding accessibility and online anaphora processing, in The 25th Annual CUNY Conference on Human Sentence Processing, New York, NY.

[B29] McElree B. (2000). Sentence comprehension is mediated by content-addressable memory structures. J. Psycholinguist. Res. 29, 111–123. 10.1023/A:1005184709695 10709178

[B30] McElree B. Foraker S. Dyer L. (2003). Memory and language memory structures that subserve sentence comprehension. J. Mem. Lang. 48, 67–91. 10.1016/S0749-596X(02)00515-6

[B31] Nairne J. S. (1990). A feature model of immediate memory. Mem. Cogn. 18, 251–269. 10.3758/BF03213879 2192233

[B32] Nicol J. Swinney D. (1989). The role of structure in coreference assignment during sentence comprehension. J. Psycholinguist. Res. 18, 5–19. 10.1007/BF01069043 2647962

[B33] Oberauer K. Kliegl R. (2006). A formal model of capacity limits in working memory. J. Mem. Lang. 55, 601–626. 10.1016/j.jml.2006.08.009

[B34] Osterhout L. Bersick M. McLaughlin J. (1997). Brain potentials reflect violations of gender stereotypes. Mem. Cogn. 25, 273–285. 10.3758/BF03211283 9184479

[B35] Parker D. Phillips C. (2014). Selective priority for structure in memory retrieval, in The 27th Annual CUNY Conference on Human Sentence Processing, Columbus, OH.

[B36] Phillips C. Wagers M. W. Lau E. F. (2011). Grammatical illusions and selective fallibility in real-time language comprehension, in Experiments at the Interfaces (Syntax and Semantics), Vol. 37, ed Runner J. T. (Bingley: Emerald Group Publishing Limited), 147–180.

[B37] R Development Core Team (2009). R: A Language and Environment for Statistical Computing. Vienna: R Foundation for Statistical Computing. ISBN: 3-900051-07-0.

[B38] Runner J. T. Head K. D. L. (2014). What can visual world eye-tracking tell us about the binding theory?, in Empirical Issues in Syntax and Semantics 10, ed Piñón C. (Paris: CSSP—Colloque de Syntaxe et Sémantique à Paris), 269–286.

[B39] Runner J. T. Sussman R. S. Tanenhaus M. K. (2006). Processing reflexives and pronouns in picture noun phrase. Cogn. Sci. 30, 193–241. 10.1207/s15516709cog0000_58 21702814

[B40] Sturt P. (2003). The time-course of the application of binding constraints in reference resolution. J. Mem. Lang. 48, 542–562. 10.1016/S0749-596X(02)00536-3

[B41] Sturt P. Kwon N. (2013). The processing of raising and nominal control, in The 26th Annual CUNY Conference on Human Sentence Processing, Columbia, SC.

[B42] Thompson C. K. Choy J. J. (2009). Pronominal resolution and gap filling in agrammatic aphasia: evidence from eye movements. J. Psycholinguist. Res. 38, 255–283. 10.1007/s10936-009-9105-7 19370416 PMC2823636

[B43] Van Dyke J. A. McElree B. (2011). Cue-dependent interference in comprehension. J. Mem. Lang. 65, 247–263. 10.1016/j.jml.2011.05.002 21927535 PMC3171743

[B44] Vasishth S. Brüssow S. Lewis R. L. Drenhaus H. (2008). Processing polarity: how the ungrammatical intrudes on the grammatical. Cogn. Sci. 32, 685–712. 10.1080/03640210802066865 21635350

[B45] Vasishth S. Lewis R. L. (2006). Argument-head distance and processing complexity: explaining both locality and antilocality effects. Language 82, 767–794. 10.1353/lan.2006.0236

[B46] Vasishth S. von der Malsburg T. Engelmann F. (2013). What eye movements can tell us about sentence comprehension. Wiley Interdisc. Rev. Cogn. Sci. 4, 125–134. 10.1002/wcs.1209 26304190

[B47] Wagers M. W. Lau E. F. Phillips C. (2009). Agreement attraction in comprehension: representations and processes. J. Mem. Lang. 61, 206–237. 10.1016/j.jml.2009.04.002

[B48] Wong T. J. Cokel E. T. Schooler L. J. (2010). An Online Database of ACT-R Parameters: Towards a Transparent Community-Based Approach to Model. Philadelphia, PA: Drexel University.

[B49] Xiang M. Dillon B. Phillips C. (2009). Illusory licensing effects across dependency types: ERP evidence. Brain Lang. 108, 40–55. 10.1016/j.bandl.2008.10.002 19007980

