# Supplementary material for: Retrieval Interference in Syntactic Processing: The Case of Reflexive Binding in English
Source: Front Psychol. 2016 May 26;7:329. doi: 10.3389/fpsyg.2016.00329 (PMC4881398; doi:10.3389/fpsyg.2016.00329)
Supplement: Supplementary file 1 [file DataSheet1.pdf]

Appendix A
The cue-based retrieval architecture

A more detailed presentation of the model is in Lewis and Vasishth (2005), Lewis et al. (2006) and Vasishth and Lewis (2006). Here we briefly describe the main features of the architecture relevant for the present discussion. The cue-based parsing theory is derived from (i) independent theory concerning the general principles of memory, as realized in the ACT-R architecture (Anderson, Byrne, Douglass, Lebiere, & Qin, 2004), (ii) parsing assumptions in psycholinguistics, and (iii) representational assumptions in theoretical syntax.

In ACT-R, cognitive processes can be defined for specific tasks by means of interactions between a declarative memory system and a procedural memory system. The declarative memory system serves as a long-term memory (semantic and episodic memory) but also serves to store the transient products of processing (in the context of parsing, this could be, for example, representations of phrases, and incremental trees). Each item in declarative memory, called a chunk, is a set of feature-value pairs. Procedural memory contains procedural knowledge specified in terms of *production rules*, which are condition–action associations.

In the cue-based retrieval architecture, lexical knowledge is stored in declarative memory, and grammatical knowledge is held in procedural memory as a set of production rules that specify how to apply the grammatical knowledge (the control structure) to incrementally parse sentences. Production rules are specified such that sentence parsing happens according to the left-corner parsing algorithm (Aho & Ullman, 1972). The novel structures constructed incrementally during sentence processing are stored in terms of chunks in the declarative memory. Each such chunk is an X-bar structure (Chomsky, 1986) representing a maximal projection with features corresponding to X-bar positions (specifier, complement, head) and other grammatical features such as person, number, gender, case and agreement. Sentence processing unfolds as a sequence of production rule firing, retrieval of memory chunks and update of the current parse tree. The parse tree is updated by creating new chunks and attaching them to the parse tree.

Apart from the symbolic system (i.e., procedural and declarative memory), the model's behavior depends on constraints imposed on the retrieval of chunks from memory. These constraints are defined in ACT-R in terms of a set of sub-symbolic computations that affect the activation of chunks. The activation value influences the retrieval probabilities and retrieval latencies of chunks. The activation fluctuates as a function of frequency, recency and prior pattern of retrievals of chunks. The total momentary activation of a chunk i is given by Equation 1, which is the sum of base level activation (B_i), the spreading activation received through retrieval cues (the first summation component), activation received due to partial match between retrieval cues and corresponding feature values in the chunks (the second summation component), and stochastic noise (ϵ).

$$A_i = B_i + \sum_{j=1}^{m} W_j S_{ji} + \sum_{k=1}^{p} P M_{ki} + \epsilon \tag{1}$$

The base-level activation of a chunk is calculated in terms of Equation 2. Here, t_j is the time since the jth successful retrieval of chunk i and d is the decay parameter.

$$B_i = \ln\left(\sum_{j=1}^{n} {t_j}^{-d}\right) \tag{2}$$

The spreading activation that a chunk i receives (the first summation component in Equation 1) is computed using W_j and S_{ji} values. W_j is typically equal to $1/m$, where m is normally the number of retrieval cues. S_{ji} is the strength of association from an element (typically a retrieval cue) j to chunk i and it is computed using Equation 3. Here, S is the maximum associative strength parameter and fan_j is the number of items associated with cue j. Associative retrieval interference arises because the strength of association from a cue is reduced as a function of the "fan" of the retrieval cue.

$$S_{ji} = S - ln(\text{fan}_j) \tag{3}$$

Activation received by means of a partial match (the second summation component in Equation 1) is computed using P and M_{ki} over p retrieval cues. P is the match scaling parameter, and M_{ki} refers to the similarity between the retrieval cue k and the corresponding value in chunk i. The range for similarity values is specified in terms of maximum similarity and maximum difference parameters. By default, similarity between a cue and a chunk is equal to the maximum similarity value if the two are the same, and is equal to maximum difference otherwise.

Finally, the mapping from activation A_i to retrieval latency T_i for a chunk i is obtained in terms of Equation 4. Here F is the scaling parameter, called the latency factor. A chunk can be retrieved only if its activation is above a certain lower limit, defined in terms of the retrieval threshold parameter (τ).

$$T_i = Fe^{-A_i} \tag{4}$$

For present purposes, what is useful about ACT-R and the cue-based retrieval theory of Lewis and Vasishth (2005) is that it provides a well-specified computational realization of the idea of memory retrieval as a noisy process of discriminating targets against a background of potentially similar distractors in short-term memory—a computational realization that has been used to build a functional (if limited) parser and applied to other sentence processing phenomena (Vasishth & Lewis, 2006; Vasishth et al., 2008).

Table A1

The list of parameter values used in the previous studies with cue-based retrieval models and the values used in the current model.

Parameter	Previous models	Current model
Noise (ϵ)	0, 0.15, 0.30, 0.45	0.30
Decay (d)	0.50	0.50
Maximum associative strength (S)	1.50	1.50
Match scale (P)	1	1
Maximum similarity	0	0
Maximum difference	-0.60	-0.60
Latency factor (F)	0.14, 0.46	0.14
Retrieval threshold (τ)	-1.50	-1.50
