# Supplementary material for: Retrieval Interference in Syntactic Processing: The Case of Reflexive Binding in English
Source: Front Psychol. 2016 May 26;7:329. doi: 10.3389/fpsyg.2016.00329 (PMC4881398; doi:10.3389/fpsyg.2016.00329)
Supplement: Supplementary file 2 [file DataSheet2.pdf]

Appendix B

Experimental items

The list consists of 24 items in four conditions that were used in the experiment. For every condition the sentence is followed by a question and the correct—Y (Yes), N (No)—answer where applicable.

1. (a) The dedicated firefighter that Henry recommended for the new job cut himself on broken glass on the floor./Did Henry cut himself on broken glass?/N.
   (b) The dedicated firefighter that Linda recommended for the new job cut himself on broken glass on the floor./Did the firefighter recommend Linda for the new job?/N.
   (c) The dedicated firefighter that Linda recommended for the new job cut herself on broken glass on the floor./Did Linda cut herself on broken glass?/N.
   (d) The dedicated firefighter that Henry recommended for the new job cut herself on broken glass on the floor./Did the firefighter recommend Henry for the new job?/N.
2. (a) The head engineer that Peter had visited in the factory convinced himself that the building was safe./Did Peter convince himself about the safety of the building?/N.
   (b) The head engineer that Nancy had visited in the factory convinced himself that the building was safe./Did the engineer visit Nancy in the factory?/N.
   (c) The head engineer that Nancy had visited in the factory convinced herself that the building was safe./Did Nancy convince herself about the safety of the building?/N.
   (d) The head engineer that Peter had visited in the factory convinced herself that the building was safe./Did the engineer visit Peter in the factory?/N.
3. (a) The professional electrician that John consulted about the wiring problems blamed himself for the fire./Did John blame himself for the fire?/N.
   (b) The professional electrician that Mary consulted about the wiring problems blamed himself for the fire./Did the electrician blame Mary for the fire?/N.
   (c) The professional electrician that Mary consulted about the wiring problems blamed herself for the fire./Did Mary blame herself for the fire?/N.
   (d) The professional electrician that John consulted about the wiring problems blamed herself for the fire./Did the electrician blame John for the fire?/N.
4. (a) The boat mechanic that Brian found in the newspaper had taught himself everything about boats from a book./Did Brian teach himself everything about boats?/N.
   (b) The boat mechanic that Jane found in the newspaper had taught himself everything about boats from a book./Did Jane teach the mechanic everything about boats?/N.
   (c) The boat mechanic that Jane found in the newspaper had taught herself everything about boats from a book./Did Jane teach herself everything about boats?/N.
   (d) The boat mechanic that Brian found in the newspaper had taught herself everything about boats from a book./Did Brian teach the mechanic everything about boats?/N.
5. (a) The famous senator that Robert volunteered for in the campaign compared himself with previous presidents./Was Robert's senator famous?/Y.
   (b) The famous senator that Melissa volunteered for in the campaign compared himself with previous presidents./Was Melissa's senator famous?/Y.
   (c) The famous senator that Melissa volunteered for in the campaign compared herself with previous presidents./Was Melissa's senator famous?/Y.
   (d) The famous senator that Robert volunteered for in the campaign compared herself with previous presidents./Was Robert's senator famous?/Y.

6. (a) The old rancher that Paul chose as a milk supplier exhausted himself after building a huge barn on the hill./Did the rancher build a barn on the hill?/Y.
   (b) The old rancher that Nina chose as a milk supplier exhausted himself after building a huge barn on the hill./Did the rancher build a barn on the hill?/Y.
   (c) The old rancher that Nina chose as a milk supplier exhausted herself after building a huge barn on the hill./Did the rancher build a barn on the hill?/Y.
   (d) The old rancher that Paul chose as a milk supplier exhausted herself after building a huge barn on the hill./Did the rancher build a barn on the hill?/Y.
7. (a) The truck driver that Frank met in the gas station hated himself for smoking so heavily./Did Frank meet any truck drivers?/Y.
   (b) The truck driver that Stacy met in the gas station hated himself for smoking so heavily./Did Stacy meet any truck drivers?/Y.
   (c) The truck driver that Stacy met in the gas station hated herself for smoking so heavily./Did Stacy meet any truck drivers?/Y.
   (d) The truck driver that Frank met in the gas station hated herself for smoking so heavily./Did Frank meet any truck drivers?/Y.
8. (a) The construction worker that David drove by on the road hit himself on the face by accident./Did the construction worker hit himself?/Y.
   (b) The construction worker that Ashlee drove by on the road hit himself on the face by accident./Did the construction worker hit himself?/Y.
   (c) The construction worker that Ashlee drove by on the road hit herself on the face by accident./Did the construction worker hit herself?/Y.
   (d) The construction worker that David drove by on the road hit herself on the face by accident./Did the construction worker hit herself?/Y.
9. (a) The tough soldier that Fred treated in the military hospital introduced himself to all the nurses.
   (b) The tough soldier that Katy treated in the military hospital introduced himself to all the nurses.
   (c) The tough soldier that Katy treated in the military hospital introduced herself to all the nurses.
   (d) The tough soldier that Fred treated in the military hospital introduced herself to all the nurses.
10. (a) The taxi driver that Andrew crashed into on the road protected himself from being hurt by steering to the side.
    (b) The taxi driver that Amelia crashed into on the road protected himself from being hurt by steering to the side.
    (c) The taxi driver that Amelia crashed into on the road protected herself from being hurt by steering to the side.
    (d) The taxi driver that Andrew crashed into on the road protected herself from being hurt by steering to the side.
11. (a) The rough lumberjack that Kevin saw on the mountain had separated himself from the outside world.
    (b) The rough lumberjack that Karen saw on the mountain had separated himself from the outside world.
    (c) The rough lumberjack that Karen saw on the mountain had separated herself from the outside world.
    (d) The rough lumberjack that Kevin saw on the mountain had separated herself from the outside world.

12. (a) The tidy janitor that Harold insulted in the school simply told himself to ignore the stupid comments.
    (b) The tidy janitor that Veronica insulted in the school simply told himself to ignore the stupid comments.
    (c) The tidy janitor that Veronica insulted in the school simply told herself to ignore the stupid comments.
    (d) The tidy janitor that Harold insulted in the school simply told herself to ignore the stupid comments.
13. (a) The rude receptionist that Melinda spoke with on the phone locked herself out of the office./Did Melinda lock herself out of the office?/N.
    (b) The rude receptionist that Melvin spoke with on the phone locked herself out of the office./Did Melvin lock himself out of the office?/N.
    (c) The rude receptionist that Melvin spoke with on the phone locked himself out of the office./Did Melvin lock himself out of the office?/N.
    (d) The rude receptionist that Melinda spoke with on the phone locked himself out of the office./Did Melinda lock herself out of the office?/N.
14. (a) The enthusiastic cheerleader that Tanya heard in the big stadium gave herself a sore throat./Was the cheerleader too quiet?/N.
    (b) The enthusiastic cheerleader that Ted heard in the big stadium gave herself a sore throat./Was the cheerleader too quiet?/N.
    (c) The enthusiastic cheerleader that Ted heard in the big stadium gave himself a sore throat./Was the cheerleader too quiet?/N.
    (d) The enthusiastic cheerleader that Tanya heard in the big stadium gave himself a sore throat./Was the cheerleader too quiet?/N.
15. (a) The respected beautician that Amy interviewed in the fashion magazine educated herself through years of hard work./Was the beautician interviewed by Amy in the newspaper?/N.
    (b) The respected beautician that Arnold interviewed in the fashion magazine educated herself through years of hard work./Was the beautician interviewed by Arnold in the newspaper?/N.
    (c) The respected beautician that Arnold interviewed in the fashion magazine educated himself through years of hard work./Was the beautician interviewed by Arnold in the newspaper?/N.
    (d) The respected beautician that Amy interviewed in the fashion magazine educated himself through years of hard work./Was the beautician interviewed by Amy in the newspaper?/N.
16. (a) The talkative cosmetician that Janice photographed for the local newspaper poisoned herself by accident./Did Janice poison herself?/N.
    (b) The talkative cosmetician that Phillip photographed for the local newspaper poisoned herself by accident./Did Phillip poison himself?/N.
    (c) The talkative cosmetician that Phillip photographed for the local newspaper poisoned himself by accident./Did Phillip poison himself?/N.
    (d) The talkative cosmetician that Janice photographed for the local newspaper poisoned himself by accident./Did Janice poison herself?/ N.
17. (a) The flight attendant that Wendy troubled on the long flight restrained herself from getting impatient./Did Wendy trouble the flight attendant?/Y.
    (b) The flight attendant that Richard troubled on the long flight restrained herself from getting impatient./Did Richard trouble the flight attendant?/Y.

(c) The flight attendant that Richard troubled on the long flight restrained himself from getting impatient./Did Richard trouble the flight attendant?/Y.

(d) The flight attendant that Wendy troubled on the long flight restrained himself from getting impatient./Did Wendy trouble the flight attendant?/Y.

18. (a) The tired hairdresser that Betsy kept at work all day drove herself home at midnight./Did the hairdresser drive herself home at night?/Y.

(b) The tired hairdresser that Ron kept at work all day drove herself home at midnight./Did the hairdresser drive herself home at night?/Y.

(c) The tired hairdresser that Ron kept at work all day drove himself home at midnight./Did Ron keep the hairdresser working until very late?/Y.

(d) The tired hairdresser that Betsy kept at work all day drove himself home at midnight./Did Betsy keep the hairdresser working until very late?/Y.

19. (a) The kindergarten teacher that Helen called for a parent-teacher meeting sent herself an email reminder./Did the kindergarten teacher send herself an email?/Y.

(b) The kindergarten teacher that Herbert called for a parent-teacher meeting sent herself an email reminder./Did the kindergarten teacher send herself an email?/Y.

(c) The kindergarten teacher that Herbert called for a parent-teacher meeting sent himself an email reminder./Did Herbert call the kindergarten teacher?/Y.

(d) The kindergarten teacher that Helen called for a parent-teacher meeting sent himself an email reminder./Did Helen call the kindergarten teacher?/Y.

20. (a) The beauty consultant that Isabelle met on the small island covered herself with ocean mud./Did the beauty consultant cover herself with ocean mud?/Y.

(b) The beauty consultant that Matthew met on the small island covered herself with ocean mud./Did the beauty consultant cover herself with ocean mud?/Y.

(c) The beauty consultant that Matthew met on the small island covered himself with ocean mud./Did the beauty consultant cover himself with ocean mud?/Y.

(d) The beauty consultant that Isabelle met on the small island covered himself with ocean mud./Did the beauty consultant cover himself with ocean mud?/Y.

21. (a) The ballet dancer that Roxanne accidentally pushed to the ground examined herself for any signs of injuries or bruises.

(b) The ballet dancer that Robby accidentally pushed to the ground examined herself for any signs of injuries or bruises.

(c) The ballet dancer that Robby accidentally pushed to the ground examined himself for any signs of injuries or bruises.

(d) The ballet dancer that Roxanne accidentally pushed to the ground examined himself for any signs of injuries or bruises.

22. (a) The eloquent feminist that Victoria collaborated with on a book defined herself as an advocate for women's rights.

(b) The eloquent feminist that Joshua collaborated with on a book defined herself as an advocate for women's rights.

(c) The eloquent feminist that Joshua collaborated with on a book defined himself as an advocate for women's rights.

(d) The eloquent feminist that Victoria collaborated with on a book defined himself as an advocate for women's rights.

23. (a) The popular matchmaker that Elizabeth met in the online chat-room described herself as a servant of Cupid.

(b) The popular matchmaker that Benjamin met in the online chat-room described herself as a servant of Cupid.

(c) The popular matchmaker that Benjamin met in the online chat-room described himself as a servant of Cupid.

(d) The popular matchmaker that Elizabeth met in the online chat-room described himself as a servant of Cupid.

24. (a) The kind caregiver that Amanda hired for the summer vacation reproached herself when the children were ill-behaved.

(b) The kind caregiver that Johnny hired for the summer vacation reproached herself when the children were ill-behaved.

(c) The kind caregiver that Johnny hired for the summer vacation reproached himself when the children were ill-behaved.

(d) The kind caregiver that Amanda hired for the summer vacation reproached himself when the children were ill-behaved.
